# Comparative Postembryonic Skeletal Ontogeny in Two Sister Lineages of Old World Tree Frogs (Rhacophoridae: *Taruga*, *Polypedates*)

**DOI:** 10.1371/journal.pone.0167939

**Published:** 2017-01-06

**Authors:** Gayani Senevirathne, Ryan Kerney, Madhava Meegaskumbura

**Affiliations:** 1 Department of Molecular Biology and Biotechnology, Faculty of Science, University of Peradeniya, Peradeniya, Sri Lanka; 2 Postgraduate Institute of Science, Faculty of Science, University of Peradeniya, Sri Lanka; 3 Department of Biology, Gettysburg College, Gettysburg, Pennsylvania, United States of America; University of Auckland, NEW ZEALAND

## Abstract

Rhacophoridae, a family of morphologically cryptic frogs, with many genetically distinct evolutionary lineages, is understudied with respect to skeletal morphology, life history traits and skeletal ontogeny. Here we analyze two species each from two sister lineages, *Taruga* and *Polypedates*, and compare their postembryonic skeletal ontogeny, larval chondrocrania and adult osteology in the context of a well-resolved phylogeny. We further compare these ontogenetic traits with the direct-developing *Pseudophilautus silus*. For each species, we differentially stained a nearly complete developmental series of tadpoles from early postembryonic stages through metamorphosis to determine the intraspecific and interspecific differences of cranial and postcranial bones. Chondrocrania of the four species differ in 1) size; 2) presence/absence of anterolateral and posterior process; and 3) shape of the suprarostral cartilages. Interspecific variation of ossification sequences is limited during early stages, but conspicuous during later development. Early cranial ossification is typical of other anuran larvae, where the frontoparietal, exoccipital and parasphenoid ossify first. The ossification sequences of the cranial bones vary considerably within the four species. Both species of *Taruga* show a faster cranial ossification rate than *Polypedates*. Seven cranial bones form when larvae near metamorphic climax. Ossification of all 18 cranial bones is initiated by larval Gosner stage 46 in *T*. *eques*. However, some cranial bone formation is not initiated until after metamorphosis in the other three species. Postcranial sequence does not vary significantly. The comparison of adult osteology highlights two characters, which have not been previously recorded: presence/absence of the parieto-squamosal plates and bifurcated base of the omosternum. This study will provide a starting point for comparative analyses of rhacophorid skeletal ontogeny and facilitate the study of the evolution of ontogenetic repatterning associated with the life history variation in the family.

## Introduction

Rhacophoridae (Old World tree frogs), are a monophyletic family [1–8] with a high diversity, constituting ca. 6% of the world’s anuran species [9]. Liem [10] analyzed the skeletal morphology in 420 lineages, representative of 14 rhacophorid genera. However, despite their prevalence and existing data for adult morphology, the patterns and processes of skeletal ontogeny is largely unknown.

Rhacophoridae has been subjected to considerable recent taxonomic revisions, resulting in the recognition of many new independent evolutionary lineages at genus level [5–6,11–16]; 11 of the 18 recognized rhacophorid genera are recent descriptions. These new generic descriptions are largely based on molecular data and to a lesser extent on molecular data and morphology [13,14,16]. One of the main impediments to new lineage identification, prior to the advent of molecular phylogenetic techniques, was the lack of consistent external morphological characters. In recent phylogenetic reconstructions of rhacophorid relationships, adult skeletal data have been sparsely used [13,14], but never skeletal ontogeny.

The evolution of the spectacular diversity of reproductive modes in rhacophorids shows that both terrestrial direct-developing and foam-nesting species arise through gel-nesting ancestors, while basal rhacophorids are aquatic breeders [15]. The terrestrial direct-developing forms, which spend their entire embryonic sequence within eggs, are placed in three well-supported clades―*Philautus*, *Pseudophilautus* and *Raorchestes*. Basal, fully aquatic-breeding, genera (*Buergeria* and *Liuixalus*) exhibit a biphasic lifecycle, i.e., eggs are deposited in water and a free-swimming larva metamorphoses into an adult. The gel-nesting species that lay terrestrial eggs with aquatic larvae are in several distinct clades (*Kurixalus*, *Mercurana*, *Gracixalus*, *Beddomixalus*, *Frankixalus*, *Feihyla*). Finally, foam-nesting genera are in two paraphyletic clades (*Rhacophorus*, *Polypedates*, *Taruga*, *Ghatixalus* in one clade and *Chiromantis* as another). These have terrestrial foam nests and postembryonic free-swimming tadpoles [2,5,6,13,15]. Skeletal ontogeny across developmental stages of these forms is not known, except for a single study on *Pseudophilautus silus* [17].

Molecular phylogenies are often used to map data such as morphology and life history traits. However, this has not been applied to many newly recognized taxa, including novel rhacophorids. While several classical studies concentrate on osteology and ontogeny to ascertain higher-level systematics [18,19], ontogeny is not used to resolve the Rhacophoridae [10,20–22]. It is only now possible to analyze the osteology of Rhacophoridae in a phylogenetic context.

Sri Lankan rhaocophorids belong to three independent evolutionary lineages representative of two major life history strategies: foam-nesting *Polypedates* and *Taruga* [14], and terrestrial direct-developing *Pseudophilautus* [2,5,6,15]. Ontogenetic skeletal development of the newly recognized genus *Taruga*, which was previously referred to as a part of *Polypedates*, has never been studied. Currently *Taruga* and *Polypedates* are recognized as sister lineages [6,13,14,15]. *Taruga* is an endemic genus, with adult morphological characters (prominent calcar at the distal end of the tibia, conical tubercles surrounding the cloaca) and tadpole morphologies (features of the buccal cavity and vent tube), distinguishing it from *Polypedates* [14].

Using an almost complete series of differentially stained tadpoles, metamorphs and adults, we examine the postembryonic skeletal development and adult skeletal osteology of the four species belonging to the two sister lineages of foam nesters: *Taruga* (*T*. *eques* and *T*. *longinasus*) and *Polypedates* (*P*. *cruciger* and *P*. *maculatus*). Here, we compare the patterns and processes of ossification in these two genera to facilitate deeper level comparative analyses of morphological evolution within Rhacophoridae.

## Materials and Methods

### Field collection and lab rearing

Four freshly deposited foam nests from two species each of *Taruga* and *Polypedates* were collected from the field: *T*. *eques–*Agarapatana, 6.901820°N, 80.690482°E; *T*. *longinasus*–Kanneliya, 6.248910°N, 80.333669°E; *P*. *cruciger*–Kurunegala, 7.488060°N, 80.363873°E; *P*. *maculatus*–Kantale, 8.351803°N, 81.004516°E. The nests, together with the substrate in which they were laid, were carefully transferred to our lab (University of Peradeniya), in sealed polyethylene bags placed in a cooler. They were positioned above aquaria containing aged tap water so that hatching tadpoles would fall directly into the water to undergo further development. The tadpoles were raised in the lab under identical water quality, light (12 hr day/night) and feeding conditions. They were fed twice per day; food uneaten 10 min after feeding was siphoned off and the water levels adjusted using aged tap water. They were sampled periodically from hatching (Gosner [23] stage 24) to a fully metamorphosed froglet (stage 46) to represent every developmental stage. Two adult males from each species were also collected from the field (same locations as above).

Research was conducted under the permission of Department of Wildlife Conservation (permit no. WL/3/2/13/13) and Forest Department (permit no. R&E/RES/NFSRC/14) of Sri Lanka. Specific methods of collection, euthanasia, tissue sampling and fixation followed the guidelines for use of live amphibians were approved by the ethical committee of Postgraduate Institute of Science, University of Peradeniya at its 16th meeting held on 14th November 2014.

### Preservation and osteology

Sampled tadpoles were euthanized using tricaine methanesulphonate (MS-222) and preserved in 10% neutral-buffered formalin. They were stored in 70% alcohol following a graded alcohol series of 30% and 55%; the specimens were kept overnight at each step. One to three larvae were taken from each stage between 25 and 46 (S1–S4 Tables). Additionally, two adults from each representative species, *Polypedates cruciger* (average snout-vent length (SVL) = 55.30 mm), *P*. *maculatus* (average SVL = 54.78 mm), *Taruga eques* (average SVL = 38.76 mm) and *T*. *loginasus* (average SVL = 38.90 mm) were also differentially stained for bone and cartilage. Osteological preparations and descriptions are based on 150 specimens (S1–S4 Tables). Specimens were cleared and differentially stained for bone and cartilage using alizarin red and Alcian blue, respectively [24]. To minimize differences in cleared and stained specimens, all the specimens were processed at the same temperature and treated with same stock solutions. Each specimen was scored for presence of bone by using a stereomicroscope within 1–3 days of the staining process (S1–S4 Tables). Ossification indices were calculated for each stage by dividing the number of bones observed at a specific stage by the total number of bones. Cartilage terminology follows [25–32].

### DNA barcoding and genetic analyses

The 16S rRNA mitochondrial gene fragment from a single tadpole from each foam nest was amplified and sequenced to ascertain species identity. Tadpoles were euthanized in MS-222, preserved in absolute ethanol and stored at –20°C in the Department of Molecular Biology & Biotechnology (DZ), University of Peradeniya. DNA was extracted from ethanol-preserved tail muscle using a standard protocol [33]. Portions of the mitochondrial 16S ribosomal RNA gene (600 bp) were amplified by PCR using primer sets 16Sar and 16Sbr [34]. PCR products were sequenced directly with dye-termination cycle sequencing. Newly generated sequences were checked using 4peaks (v. 1.7.1).

Published sequences from 37 closely related species (S5 Table) of *Polypedates* and *Taruga* (following Li *et al*. [5,6] Meegaskumbura *et al*. [13]) were included in a dataset. Additionally, four mantellid species were used as the outgroup (S5 Table). The compiled 16S rRNA dataset was aligned using ClustalW as implemented in MEGA v. 6.0 [35]. Uncorrected pairwise distances were calculated using PAUP* 4.0b10 [36] (S6 Table). Highly variable regions were manually removed from the dataset; the final dataset consisted of 465 bp. The best-fit model (GTR+I+G) was chosen using jModeltest v. 2.1.4 [37]. Maximum likelihood (ML) analysis was performed to infer relationships among the lineages and clades using the software GARLI (Zwickl 2006) on the Cipres Science Gateway.

## Results

### Phylogenetic position

**Fig 1:**
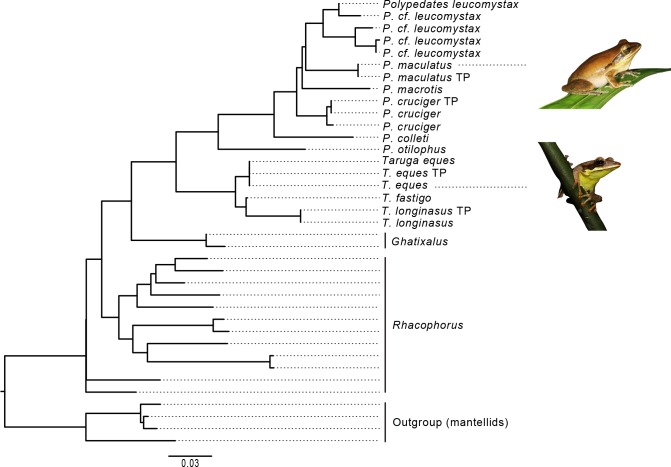
The 16S Maximum Likelihood phylogram of the closest lineages of *Polypedates* and *Taruga*, represented by 33 taxa. Four mantellid species are used as the outgroup. Tadpole sequences are indicated as “TP.”

Phylogenetic relationships among the rhacophorid taxa presented here, agree with previous analyses [6,13,15]. Tadpole sequences of a given species cluster with their respective adult sequences, forming two strongly supported clades (*Taruga* and *Polypedates*); uncorrected pairwise distances between the tadpole sequence (GenBank accession numbers: KY111847–KY111850; given upon acceptance) and its respective adult sequence range between 0.001–0.002 (Fig 1; S6 Table).

### Larval neurocranium and the first oropharyngeal arch (stage 35)

A nearly complete ontogenetic series of tadpoles was examined for each of the four species, which represent two genera (S1–S4 Tables). Description of the neurocranium and first oropharyngeal arch at stage 35 is followed by descriptions of cranial and postcranial bones.

**Fig 2:**
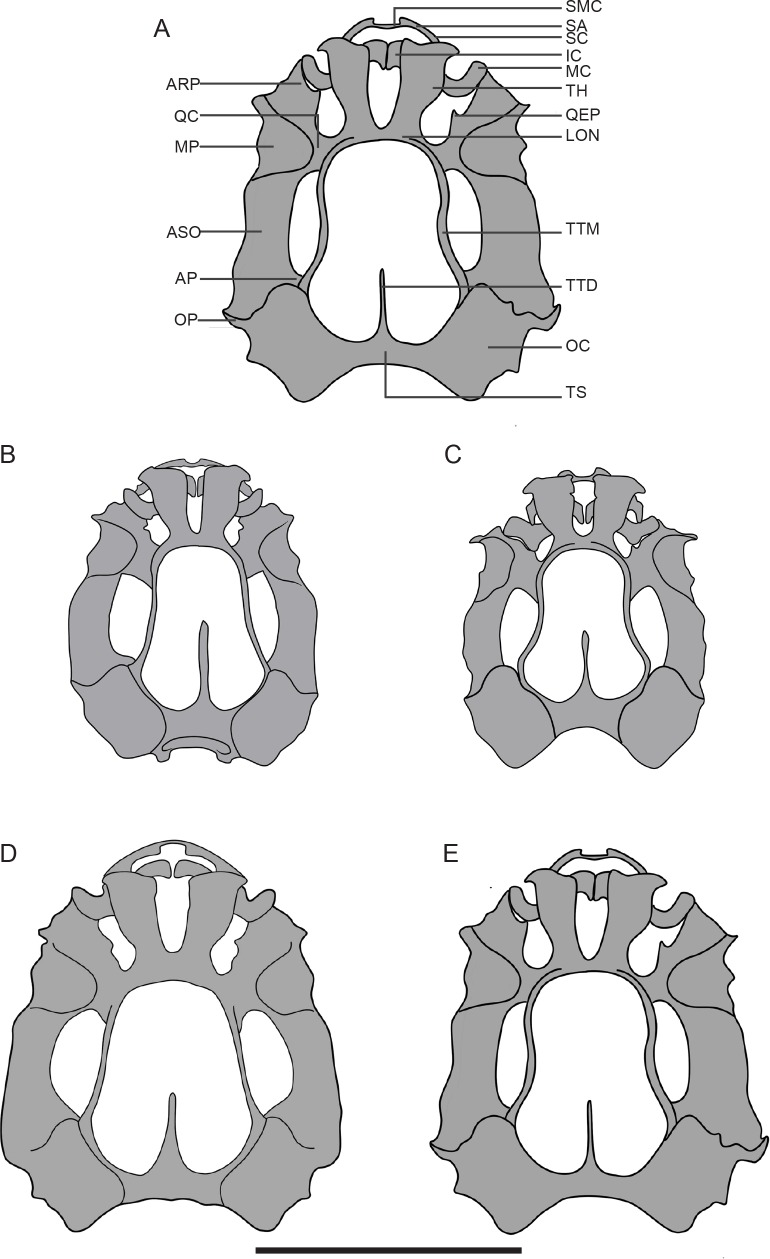
Chondrocrania of the foam-nesting genera, *Polypedates* and *Taruga*. (A) A labeled chondrocranium of *Polypedates maculatus*. Comparative illustrations of the chondrocrania of the four species: *Taruga eques* (B), *Taruga longinasus* (C), *Polypedates cruciger* (D) and *Polypedates maculatus* (E). Abbreviations: AP, ascending process; ARP, articular process; ASO, arcus subocularis; IC, infrarostral cartilage; LON, lamina orbitonasalis; MC, Meckel’s cartilage; MP, muscular process; OC, otic capsule; OP, otic process; QC, anterior quadratocranial commissure; QEP, quadratoethmoidal process; SA, suprarostral ala; SC, suprarostral cartilage; SMC, suprarostral medial corpus; TH, trabecular horns; TTD, taenia tecti medialis; TTM, taenia tecti marginalis; TS, tectum synoticum. Scale bar: 5 mm.

The neurocranium is slightly longer than wide in each species. The neurocranium is widest at the midpoint of the arcus subocularis in *Taruga eques* and *T*. *longinasus* but at the posterior end of the arcus subocularis in *Polypedates cruciger* and *P*. *maculatus* (Fig 2). The chondrocranium of each species is oval-shaped in dorsal view.

#### Ethmoid region

In all four species the trabecular horns diverge laterally from one another and form ligamentous attachments to the lateral alae of the suprarostral cartilages. Anterior ends of the trabecular horns are almost flat. Trabecular horns account for 32.2% of chondrocranial length in *T*. *eques*, 26.7% in *P*. *cruciger*, 31.5% in *T*. *longinasus* and 29.5% in *P*. *maculatus*. The lamina orbitonasalis is present anterior to the quadratocranial commissure. The nasal septum is absent.

#### Braincase

All four species have a single large opening (i.e. frontoparietal fontanelle) in the roof of the larval braincase. This opening is demarcated anteriorly by the lamina orbitonasalis, posteriorly by the tectum synoticum, and laterally by the otic capsules and taenia tecti marginalis. The taenia tecti medialis subdivides the posterior frontoparietal fontanelle into two lateral halves. The taenia tecti medialis is almost 50% as long as the frontoparietal fenestra except in *P*. *maculatus* (*T*. *eques*, 46%; *T*. *longinasus*, 41%; *P*. *cruciger*, 43%; and *P*. *maculatus*, 37%). Two pairs of craniopalatine and primary carotid foramina are present ventrally (not shown).

#### Otooccipital region

Each oval-shaped otic capsule possesses a large fenestra ovalis ventrolaterally. The prootic and oculomotor foramina are visible in the orbital cartilage; the latter opening is smaller and directed more ventrally. Laterally projecting crista parotica can be seen anterolateral to the otic capsules. The crista parotica anteriorly bears a well-developed anterolateral process, which is long, finger-like in *T*. *longinasus*, stout and triangular in *T*. *eques* and long and triangular in *P*. *cruciger* and *P*. *maculatus* (Fig 2). A laterally projecting, triangular posterolateral process is well developed in the two *Polypedates* species but reduced in the two *Taruga*. Paired occipital arches extend ventrally from the posteromedial margins of the otic capsules and form occipital condyles by fusing with the basal plate.

#### Palatoquadrate cartilage

The palatoquadrate lies lateral to the braincase; it is oriented anteroposteriorly and parallel to the longitudinal axis of the chondrocranium. The anterior quadratocranial commissure, the ascending process and the otic process connect the palatoquadrate to the neurocranium. The posterior curvature of the palatoquadrate is at the level of attachment to the ascending process of orbital cartilage. The quadratoethmoidal process is triangular extending from the anterior margin of the anterior quadratocranial commissure. The processus pseudopterygoideus is absent in all four species. The muscular and articular processes constitute the anterior processes of the palatoquadrate. The muscular process is dorsally expanded; it is 1.74 mm wide in *P*. *cruciger*, 1.50 mm in *P*. *macualtus*, 1.26 mm in *T*. *eques* and 0.94 mm in *T*. *longinasus*.

#### Suprarostral cartilages

Paired suprarostral cartilages are oriented perpendicular to the longitudinal axis of the chondrocranium and lie between the trabecular horns. They support the upper horny beaks. Each comprises a flat, rectangular ala laterally and a medial corpus. Adjacent corpora are fused ventromedially in *T*. *longinasus*, *T*. *eques* and *P*. *maculatus*, and dorsomedially in *P*. *cruciger*. The gap between posterior margins of the medial corpus is 6.8% of chondrocranial width in *P*. *cruciger*; the gap between anterior margins of the medial corpus is 11.5% in *T*. *longinasus*, 9.3% in *T*. *eques* and 6.0% in *P*. *maculatus*. Lateral alae curve rostrally, where they fuse with the medial corpus. The suprarostrals of *T*. *longinasus*, *T*. *eques* and *P*. *maculatus* appear U-shaped in anterior view but assume an inverted U-shape in *P*. *cruciger*.

#### Lower jaw

Paired Meckel’s and infrarostral cartilages form the lower jaw. Infrarostrals are wedge-shaped and triangular in cross section. Each transverse infrarostral cartilage is confluent with each other medially via a thin bar of cartilage (commissura intramandibularis). The posterior margins of each infrarostral are connected via a ligament with the anterior margin of the adjacent, sigmoid-shaped Meckel’s cartilage. Meckel’s cartilage articulates with the articular process of the palatoquadrate laterally.

### Ossification of the skull

**Fig 3:**
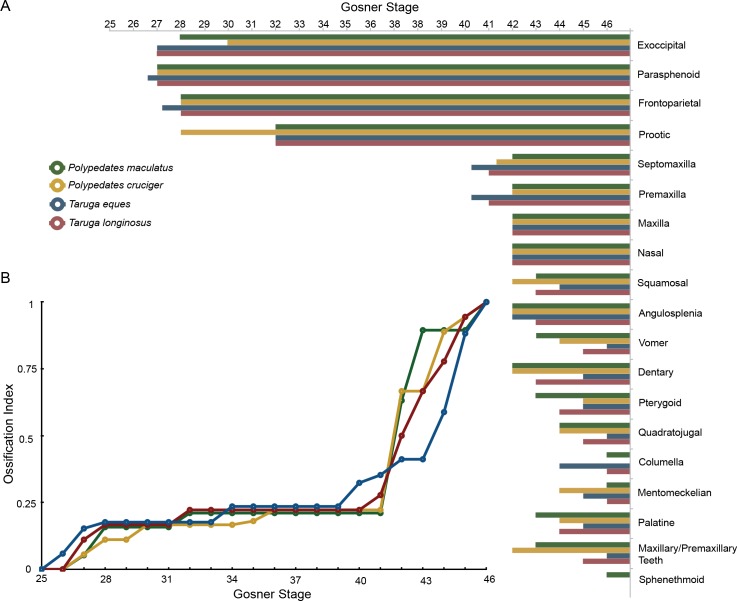
Comparison of ossification sequences and ossification indices (cranial bones) of *Taruga* and *Polypedates*. (A) Initial appearance of cranial bones (*N* = 19) is plotted against the Gosner stage for the four species. (B) Ossification indices of the four species is calculated to each individual (circles) by dividing the number of present ossified skull bones by the total number of scored elements in the cranium. The initial ossification is relatively slow in the two species of *Polypedates* (green and yellow open dots), fast in the two species of *Taruga* (blue and red open dots). But as development progresses, ossification rate of *Polypedates* species increases.

The cranial ossification sequence is depicted in Fig 3. The following comparisons of adult bones among the four species emphasize phylogenetically informative characters (character matrix is given as S11 Table) recognized by Liem [10] and Scott [38].

#### Parasphenoid

The parasphenoid is a triradiate, medial and dorsoventrally flattened bone that invests the basal plate. It is the first bone to ossify in all four species. Ossification begins in the cultriform process but continues posteriorly along the midventral floor of the braincase to the exoccipital region, where it extends laterally to form the paired alae. The anterior, bifid tip of the cultriform process lies posterior to the planum antorbitale; it is serrated in adult *T*. *eques* and *T*. *longinasus* and sharply pointed in *P*. *cruciger* and *P*. *maculatus*. The alae extend laterally and are moderately long; they are 45.9% of cranial width in *P*. *cruciger*, 37% in *P*. *maculatus*, 46.2% in *Taruga eques* and 67.1% in *T*. *longinasus*. The caudal edge of the sphenethmoid articulates with the rostral edge of the parasphenoid seen at stage 46 in *P*. *maculatus* but only in adults in *P*. *longinasus*, *T*. *eques* and *P*. *cruciger*. Alae fuse with the otic capsules at stage 44 in *T*. *eques*, stage 45 in *T*. *longinasus*, stage 42 in *P*. *cruciger* and stage 46 in *P*. *maculatus*.

#### Exoccipitals

Ossification of the paired exoccipitals begins along the dorsal regions of the occipital condyles. It continues around the middle part of the occipital arch dorsally and ventrally, and also along the otic capsules and basal plate. Subsequently, ossification extends laterally into the posteromedial portions of the otic capsules, the occipital condyles and the margins of the jugular foramen. Exoccipitals and prootics fuse to form the posterolateral parts of the braincase and the anterolateral, anteroposterior and anteromedial margins of the otic capsules at stage 44 in *Taruga eques*, *T*. *longinasus* and *P*. *cruciger*, and at stage 43 in *P*. *maculatus*,

#### Frontoparietals

Ossification begins near the midpoint of the taenia tecti marginalis and extends rapidly along the longitudinal axis, growing anteriorly and posteriorly. The frontoparietal ossification also proceeds medially at a comparatively slower rate, thus increasing in length and breadth while flanking the frontoparietal fenestrae. In adults, frontoparietals are slender, long, paired bones, which are narrowly separated at the midline. The frontoparietal fontanelle is bordered by taenia tecti marginalis laterally, nasal cartilages anteriorly and sphenethmoid anterolaterally. The greatest width of the skull (in adults) is achieved posterior to the otic capsules in all four species. Only the two *Polypedates* species possess parieto-squamosal plates, which are placed laterally on the posterolateral ends of the frontoparietals.

#### Prootics

Osteogenesis of prootics is initiated as a small center along the anteromedial margin of the otic capsule. These bones are deposited gradually, laterally and posteriorly over the anterior and anterolateral margins of the otic capsules. Prootics articulate with the lateral margins of frontoparietals dorsally (at stage 43 in *T*. *eques*, stage 45 *T*. *longinasus*, stage 44 in *P*. *cruciger* and stage 42 in *P*. *maculatus*). In adults, prootics form the anterolateral and ventrolateral margins of the otic capsules, and posterolateral walls of the braincase.

#### Septomaxilla

Osteogenesis of the septomaxillae is initiated as tiny centers, located anterolaterally within the nasal capsules (see S1–S4 Tables for ossification sequences of each individual species). In adults, paired, dermal, semilunar-shaped septomaxillae lie within the nasal capsules, below the nasal roof, supporting the external nares. Septomaxillae are clearly visible between the nasals and pars facialis of the maxilla in lateral view, and also can be observed between the oblique cartilage and nasals in the dorsal view.

#### Premaxilla

Initial ossification of the alary process is visible dorsal to the trabecular horns at stage 40 in *T*. *eques*, stage 41 in *T*. *longinasus* and stage 42 in both *P*. *cruciger* and *P*. *maculatus*. Dentary and palatine processes of the premaxillae appear next, respectively. Premaxillary teeth arise as short, pointed buds at stage 42 in *P*. *cruciger* (number of teeth, left/right: 6/5) and at stage 46 in *P*. *maculatus* (8/8) but after metamorphosis in *T*. *longinasus* and *T*. *eques*. After metamorphosis, premaxillae unite syndesmotically, completing the upper jaw anteriorly. The dentary process of the premaxilla bears a horizontally oriented dental ridge at stage 46 in all four species. The alary process reorients vertically during metamorphosis, expanding dorsally when the trabecular horns erode during metamorphosis. In adults, paired premaxillae complete the maxillary arcade anteriorly. The premaxillae are well separated from one another anteriorly and also from the laterally adjacent maxillae. The premaxillae are located anteromedially, and lay dorsal to the proximal trabecular horns. These bones are composed of dentary, alary and palatine processes. The alary process of the premaxilla is curved laterally and support the cartilage of the nasal capsules. The palatine process is posteromedially oriented, and serves as a site for attachment of the soft tissue lining the buccal cavity.

#### Maxilla

**Fig 4:**
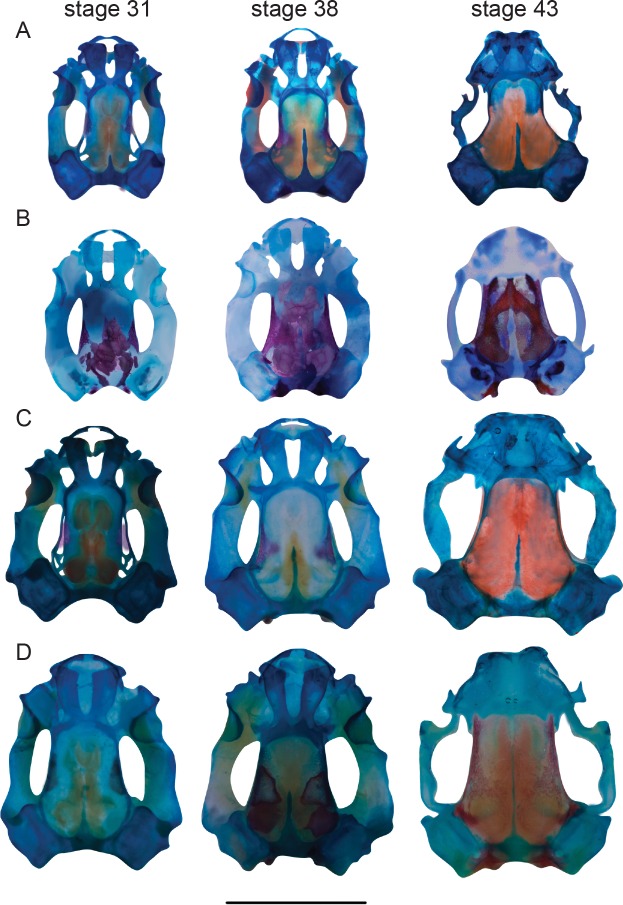
Modification and repatterning of the chondrocrania at stages 31, 38 and 43 of *Taruga longinasus* (A), *T*. *eques* (B), *Polypedates maculatus* (C), and *P*. *cruciger* (D).

The maxillae are paired, dermal, dentigerous bones containing dentary, palatine and facial processes. Initial thin ossifications (just posterior to septomaxillae in dorsal view) are located on either side of the skull along the posterior margin of the suprarostral cartilages. These rapidly extend anteriorly and posteriorly to form the pars facialis. The maxillary ossification reaches the level of the posterior margin of the orbit by stage 43 in all species. The facial process articulates with the premaxilla, forming a pointed snout in *T*. *eques* and *T*. *longinasus* and a blunt snout in both *Polypedates*. Premaxillae and maxillae begin to overlap at stage 44 in *T*. *eques*, stage 45 in *T*. *longinasus*, stage 46 in *P*. *maculatus* and in adults in *P*. *cruciger* (Fig 4). In *T*. *eques*, the maxilla extends beyond the caudal margin of the eye at stage 45 and it articulates with the quadratojugal at stage 46. This articulation also occurs at stage 46 in *P*. *maculatus and T*. *longinasus*, but it is seen in adults in *P*. *cruciger*. The facial process of the maxilla articulates broadly with the quadratojugal. Maxillary teeth are first visible at stage 42 in *P*. *cruciger* (number of maxillary teeth: 9/8), at stage 46 in *P*. *maculatus* (12/13) and in adults in *T*. *longinasus* (12/12) and *T*. *eques* (11/13).

#### Nasals

The nasals are paired, crescent shaped, expansive bones roofing part of the nasal capsules. The long axes are arranged transversely, parallel to the maxillae in all four species. After the formation of cartilaginous tectum nasi, paired centers of ossifications are narrowly separated from one another. This separation occurs anteriorly to the frontoparietals on the dorsal borders of the nasal capsules. The long, tapering maxillary process of the nasal extends ventrolateral, and adjoins facial process of maxilla in adults in all four species. The crescent shape of the nasals is maintained by equal rates of ossification occurring anteromedially and anterolaterally, widening along the anteroposterior axes. Nasals do not articulate medially, or with the frontoparietals posteriorly or the sphenethmoid anteriorly. In later stages (stages 44, 45, 46 in all four species) the anteromedial tip of the nasal grows and stops abruptly. Nasals are not completely developed by stage 46 in all four species, but nasals overlap the palatines in all adult specimens and have a more lateral orientation. In adults, nasals do not overlap either the sphenethmoid or each other.

#### Angulosplenials and dentaries

The mandible comprises three paired bony elements: angulosplenial, dentary and mentomeckelian. The angulosplenials are dermal bones that occupy posterior and anterior regions of the lower jaw. They do not articulate with the dentaries and mentomeckelians. Osteogenesis is initiated along the center of the ventral side of transversely oriented Meckel’s cartilage. Ossification proceeds along the ventral and lingual sides. The angulosplenials approach the mandibular articulation posteriorly and invest the lingual surface anteriorly after completion of metamorphosis. In adults, the coronoid processes can be observed in the dorsomedial portion of the posterior end of angulosplenials. The dentary is a dermal, small, dentate, slender bone investing anterolateral and external surfaces of the lower jaw. The dentaries appear as long, thin ossifications associated with the straightening and fusion of the infrarostrals with Meckel’s cartilages. Initial ossifications appearing along the fused margin of the infrarostrals and Meckel’s cartilage proceeds posteriorly retaining a medial separation. The dentary fuses to the mentomeckelians after metamorphosis.

#### Mentomeckelians

The mentomeckelians are small, paired endochondral bones that occupy the anteromedial parts of the mandible. Their initial ossification appears on the ventral surface of the infrarostrals. In all four species, at stage 46, mentomeckelians remain cartilaginous at their medial tips, appearing neither fused nor articulated.

#### Columella

A long, laterally oriented columella appears between the fenestra ovalis of the exoccipital and the otic ramus of the squamosal. The proximal footplate and the basal part (pars media plectri) of the columella are faintly ossified (see S1–S4 Tables for specific stages). However, ossification of the columella can be only seen in adults of *P*. *cruciger*.

#### Palatine

The palatines are paired, elongated, edentate, slim, and transversely oriented dermal bones that lie perpendicular to the longitudinal axis of the skull. They occupy the ventral side of the antorbital process, between the maxilla and the sphenethmoid, posterior to the paired vomers. Initial ossification is observed along the anterior margin of the planum antorbitale (see S1–S4 Tables for specific stages). As growth proceeds, the palatines thicken and elongate. They are curved dorsally and extend while fusing with the sphenethmoid anteriorly (fusion of the palatines with the sphenethmoid is seen in adult specimens of *P*. *cruciger*, *T*. *eques* and *T*. *longinasus;* but observed at the stage 46 in *P*. *maculatus*) while terminating bluntly.

#### Squamosal

Dermal, paired squamosals invest the cartilaginous palatoquadrate. They are comprised of three rami: ventral, zygomatic and otic. The ventral ramus is the largest of the three. It appears as a small ossification on the anterior margin of the larval muscular process. During the metamorphic reorientation of the palatoquadrate, the squamosal moves from anterior to the orbit to a position lateral to the otic capsule. The final orientation is achieved by stage 44 in *T*. *eques*, stage 43 in *T*. *longinasus*, stage 42 in *P*. *cruciger* and stage 43 in *P*. *maculatus*. The squamosals articulate distally with the quadratojugal at stage 46 in both *Taruga* species, and postmetamorphically in both *Polypedates* species. The short, small zygomatic ramus, projects anterodorsally from the ventral shaft of the squamosal and articulates with facial process of maxilla. Otic ramus ossifies concomitantly with the zygomatic ramus at stage 46 in *T*. *eques* and *T*. *longinasus*, and in adults in *P*. *cruciger* and *P*. *maculatus*; the otic ramus has a slight posterodorsal orientation, lying laterally adjacent to the anterolateral corner of the cartilaginous crista parotica. The squamosal arms rapidly elongate to form triradiate T-shaped bones in adults.

#### Pterygoid

The pterygoids are one of the ventral components of the suspensorium. These robust, dermal, triradiate bones have anterior, posterior and medial rami. The initial ossifications are observed along the ventromedial surface of the articular process of the palatoquadrate. As larvae grow, ossifications continue along the anterior and posterior margins. Ossifications along the ventromedial surface of the cartilaginous pterygoid process give rise to the long anterior ramus, which terminates near the anterior margin of the orbit. The medial rami, which are much shorter in a dorsomedial direction, extend onto the ventrolateral margin of the otic capsule. The bones are triradiate after the three rami expand in both *Taruga* species at stage 46 and in adults in both *Polypedates*.

#### Vomer

The vomers form as tiny, paired ossification centers at the posteromedial corners of the internal nares. Prechoanal, postchoanal and anterior processes make up the lateral, posterior and anterior components of the vomers, respectively. The prechoanal process forms the anterior margin of the choana. The postchoanal processes appear after metamorphosis in all four species. They support the anteromedial margins of the choana. Initial ossifications underlying the nasal capsules expand rapidly lateral to the anterior tip of the parasphenoid. Vomerine teeth are seen in adults of *T*. *eques* and *P*. *cruciger*. However, vomerine teeth are not distinct in the stained adult specimens of *T*. *longinasus* and *P*. *maculatus*.

#### Quadratojugal

The quadratojugals are paired, slender, dermal bones. Laterally, the anterior ends of the quadratojugals overlap the posterior ends of maxillae, completing the maxillary arcade ventrally (at stage 46 in *T*. *eques* and in adults in the remaining three species). The posterior ends of the quadratojugal ossify on the ventrolateral surface of the articular process of the palatoquadrate. These ends articulate with the ventral ramus of the squamosals. Further bone development occurs by lengthening of the bones, where the quadratojugal, maxillae and squamosal are interconnected at metamorphosis.

#### Sphenethemoid

**Fig 5:**
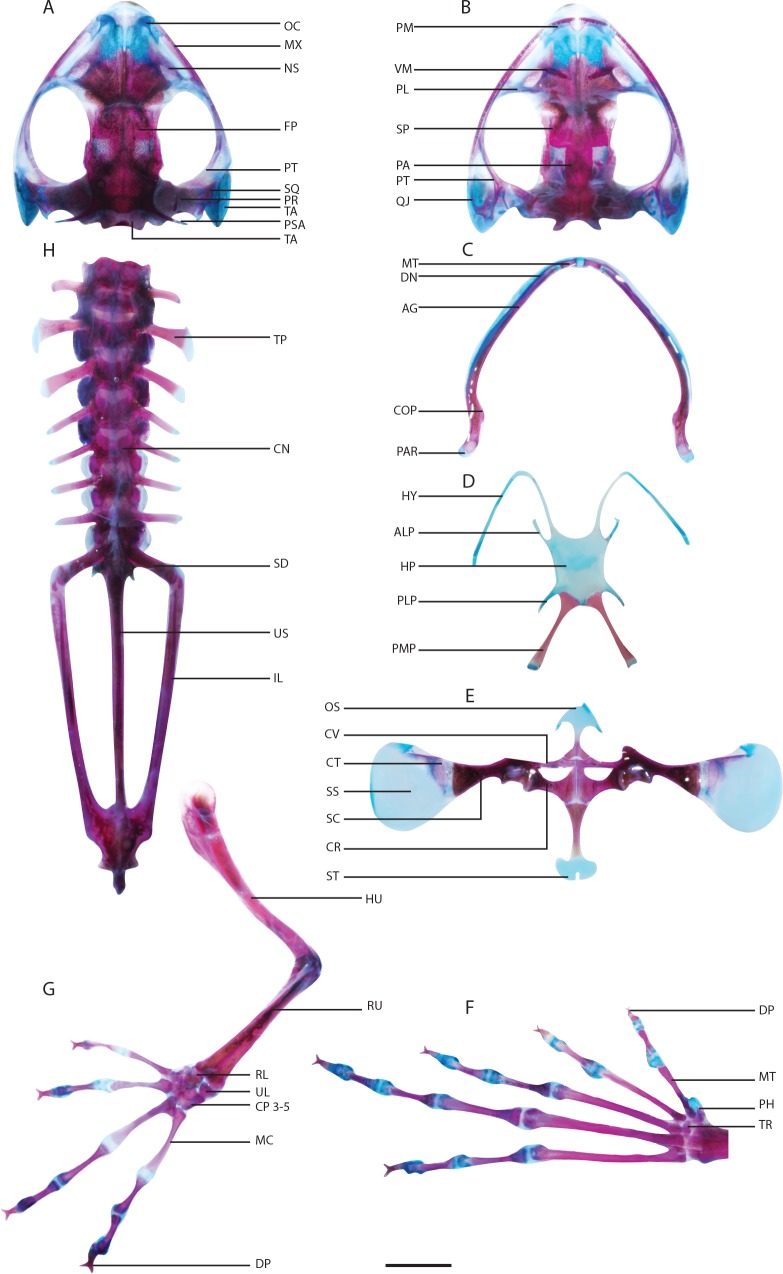
Osteology of *Polypedates cruciger*, adult male. (A) Cranium, dorsal view. (B) Cranium, ventral view. (C) Lower jaw. (D) Hyoid skeleton. (E) Pectoral girdle. (F) Left hind limb. (G) Left forelimb. (H) Axial skeleton. Abbreviations: AG, angulosplenial; AT, atlas; ALP, anterolateral process; CN, centrum; COP, coronoid process; CP, carpals; CR, coracoid; CT, cleithrum; CV, clavicle; DP, distal phalange digit; DT, dentary; EX, exoccipital; FP, frontoparietal; HU, humerus; HP, hyoid plate; HY, hyale; IL, illium; MC, metacarpal; MNT, mentomeckelian; MT, metatarsal; MX, maxilla; NS, nasal; OC, oblique cartilage; OS, omosternum; PA, parasphenoid; PAR, pars articularis; PH, prehallux; PL, palatine; PLP, posterolateral process; PM, premaxilla; PMP, posteromedial process; PR, prootic; PT, pterygoid; QJ, quadratojugal; RL, radiale; RU, radioulna; SC, scapula; SD, sacral diapophysis; SP, sphenethmoid; SQ, squamosal; SS, suprascapula; ST, sternum; TA, tympanum annulus; TP, transverse process; TR, tarsal; UL, ulnare; US, urostyle; VM, vomer. Scale bar: 5 mm.

**Fig 6:**
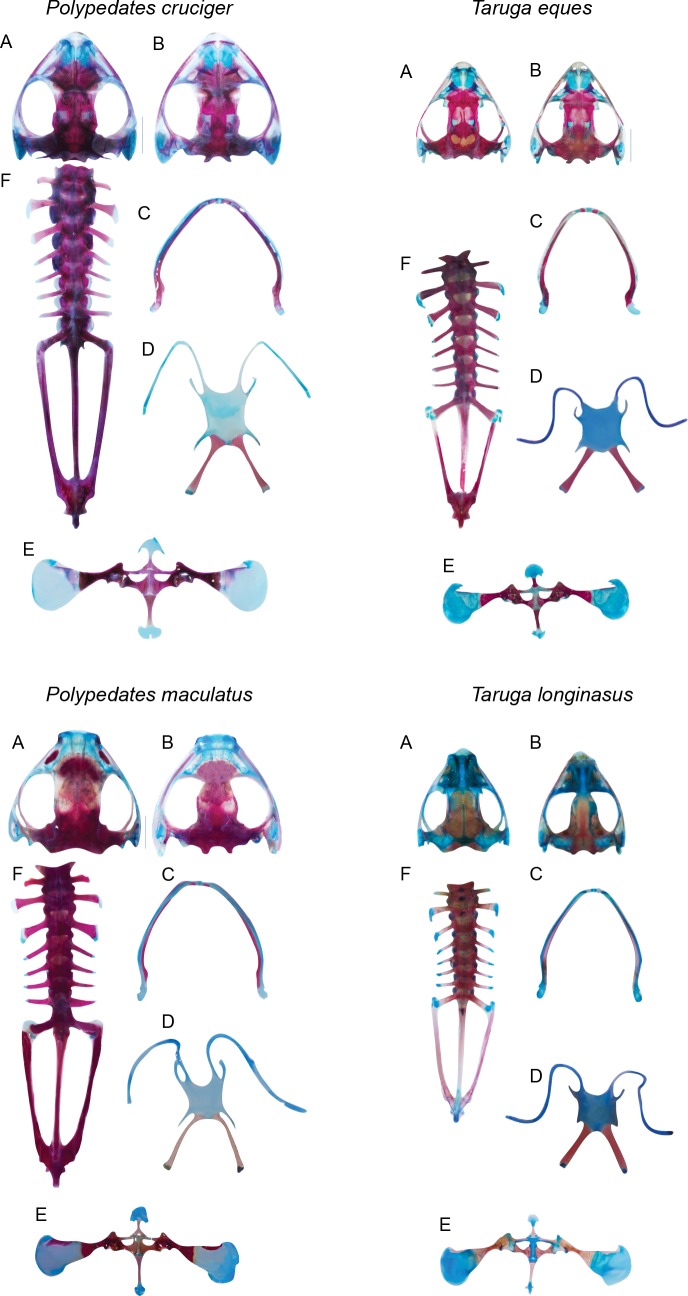
Comparison of the osteology of *Polypedates* and *Taruga*, adult males. (A) Cranium, dorsal view. (B) Cranium, ventral view. (C) Lower jaw. (D) Hyoid skeleton. (E) Pectoral girdle. (F) Axial skeleton of *Polypedates cruciger*, *P*. *maculatus*, *Taruga eques* and *T*. *longinasus*.

The sphenethmoid is an endochondral bone that contributes to the anterior part of the braincase, placed between the posterior margins of the nasal capsules and the anterior margins of the frontoparietals. The sphenethmoid originates as two centers of semi-lunar-shaped ossifications, in faint red color lateral to the anterior most part of the frontoparietals, at stage 46 in *P*. *maculatus* and in adults in *T*. *longinasus*, *P*. *cruciger and T*. *eques*. Subsequently, ossification proceeds dorsoventrally forming a deeply concave sphenethmoid anteriorly and posteriorly. Dorsally the bones are nearly covered by the frontoparietals and ventrally invested by the cultriform process of the parasphenoid (Figs 5 and 6).

### Development of the hyoid skeleton

The four species possess well-developed hyobranchial skeletons by stage 44 with thin hyoid plates possessing pairs of hyales, anterolateral (“alary process” [10]), posterolateral and posteromedial (“thyrohyal” [10]) processes. Osteogensis of the posteromedial processes are initiated by stage 45, in all four species, along the center of the cartilaginous shaft and extends along the anteroposterior axis. Adults of *T*. *eques*, *T*. *longinasus*, *P*. *cruciger* and *P*. *maculatus* possess well-ossified posteromedial processes with cartilaginous epiphyses on the distal ends and blade-like anterolateral processes (Figs 5 and 6).

### Development of the axial skeleton

The axial skeleton is composed of three regions: presacral, sacral and postsacral. There are eight presacral vertebrae (I–VII procoelous, VIII amphicoelous). The sacrum and sacral diapophyses make up the sacral region whereas the post-sacral region consists of the urochord and hypostyle. Each post-atlas vertebra consists of a cylindrical centrum (oval in a cross section), dorsal neural arch and three pairs of processes: prezygapophyses (at the anterior end), postzygapophyses (at the posterior end) and transverse processes (expanding laterally from the pedicels). The atlas lacks transverse processes and prezygapophyses in all four species and the articulation with the occipital condyles of the skull is via a pair of atlantal condyles. The ossification of neural arches begins in a single center (see S1–S4 Tables for specific stages for the four species). This center enlarges into a vertical sheet of bone that later has three components including a lamina, pedical and a pair of lateral processes. The transverse processes appear as lateral ossifications in all species, later proceeding further laterally from the center of ossification while maintaining cartilaginous tips. The transverse processes of ΙΙΙ and ΙV are the most well developed of all four species (see S8 Table). Completion of the ossification of transverse processes in all four species occurs at stage 41. The distal end of the sacral diapophyses articulates with the ilial shaft of the pelvic girdle.

### Development of the pectoral girdle

Prior to ossification, each half of the pectoral girdle is composed of the cartilaginous primordia of the scapula, suprascapula and coracoid. The scapula ossifies along its longitudinal axis, expanding laterally and articulating with the suprascapula (see S9 Table). Ossification of the suprascapula is limited to the anterior region; this gives rise to the cleithrum, which has a wider base adjacent to scapula, where it extends narrowly along the distal margin. The clavicle appears as a thin ossification along the anterior margin of the cartilaginous procoraccoid, whereas the coracoid, an endochondral bone, ossifies along the mid portion of the cartilaginous primordia of the coracoid. Ossification of these two bones is observed concurrently in all four species. The clavicle and coracoid extend laterally along their cartilaginous parts without merging or articulating with other bones.

The epicoraccoid is a cartilaginous arch that adjoins the two halves of the pectoral girdle. It appears at stage 37 in *P*. *cruciger*, *P*. *maculatus* and *T*. *eques* and in stage 36 in *T*. *longinasus*. The cartilaginous bridge joins the two halves of the pectoral girdle at stage 41 in all four species. The omosternum and sternum form anteriorly and posteriorly to this cartilaginous bridge, respectively; these two bones are ossified at stage 46 in *T*. *eques*, *T*. *longinasus* and *P*. *macualtus*, whereas in *P*. *cruciger* ossification occurs at stage 45. In adults, the base of the omosternum is clearly forked in both species of *Polypedates* but not in *Taruga* (Fig 6). Furthermore, the cartilaginous distal ends differ considerably among the four species (Fig 6). The adult sternum (“metasternum”; Liem 1970) has a bony stylus and a cartilaginous distal end (forked in *P*. *cruciger*; Figs 5 and 6)

### Development of the forelimb

**Fig 7:**
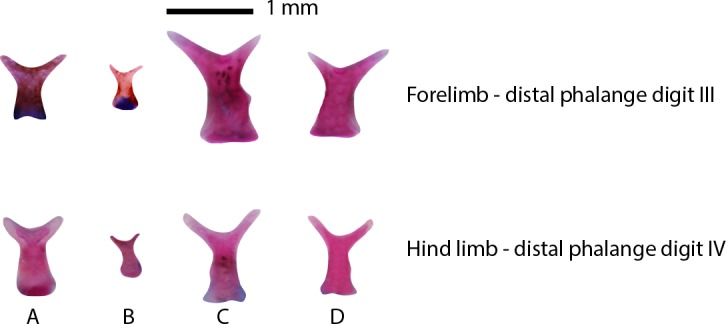
Comparison of the distal phalanges of the forelimb and hind limb. (A) *Taruga eques*. (B) *T*. *longinasus*. (C) *Polypedates cruciger*. (D) *P*. *maculatus*.

The forelimb consists of the cartilaginous primordia of the humerus, radius, ulna, radiale and ulnare at stage 36 in *P*. *cruciger* and at stage 34 in *T*. *eques* and at stage 35 in *T*. *longinasus* and *P*. *cruciger* (see S9 Table). Ossification begins in the center of the midline of the cartilaginous humerus primordium, and extends anteriorly and posteriorly. The radius and ulna ossify separately, later fusing proximally (stage 38 in all four species), distally and medially (stage 42 in all four species) to form the radioulna. Cartilaginous primordia of the proximal phalanges are present at stage 37 in *P*. *cruciger* and at stage 35 in *T*. *eques*, *T*. *longinasus* and *P*. *maculatus*. Ossifications of all the forelimb elements initiate along the mid portion and expand laterally. The phalangeal formula of all species is 2-2-3-3, where the proximal phalanges ossify first and the distal phalanges last. The ossification of the carpals can be observed only in adult specimens of *T*. *eques*. Distal phalanges are Y-shaped in all four species (Fig 7). Forelimb phalanges tend to ossify before those of hind limbs.

### Development of the pelvic girdle

The ilium, ischium and pubis unite to form the pelvic girdle. The ilium articulates with the ventral surface of the well-expanded sacral diapophysis of the axial skeleton. As development progresses the iliac shaft begins to ossify along with the mid portion of the humerus in all four species (see S10 Table). The ossifications proceed anteriorly and posteriorly along its longitudinal axis.

### Development of the hind limb

In all four species, ossification of the hind and forelimbs start concurrently (S1–S4 Tables). The femur, being the proximal element of the hind limb, articulates with the pelvic girdle. This bone along with tibia, fibula, fibulare, tibiale, metatarsals, tarsals and phalanges form the hind limbs. The tarsal region consists of the prehallux, element Y and tarsals 2–3. Proximal to the tarsal region, fibulare and tibiale can be seen, which are fused at its proximal and distal ends. The phalanges ossify also from proximal to distal. The final phalangeal formula of the hind limb is 2-2-3-4-3 in all species. The ossifications of the tarsals were not observed even at stage 46, and considered as postmetamorphic bones in all four species of *Taruga* and *Polypedates*.

## Discussion

This comparison of the skeletal morphology of the two closely related foam-nesting lineages highlights phylogenetically informative characters at two different levels: between species and between genera. Adult osteology and chondrocranial morphology of the rhacophorids have been examined in several studies [10,13, 14,18,19,20–22,38]; however, little information is available on the skeletal development [39–42,17].

The four species differ in chondrocranial morphology. *Polypedates cruciger* and *P*. *maculatus* have larger chondrocrania than *Taruga eques* and *T*. *longinasus*. There are substantial differences between the genera in the shape of the anterolateral processes and presence/absence of posterolateral processes on the otic capsules. Furthermore, the inverted U shape of the suprarostral cartilage is considerably different in *P*. *cruciger* when compared with upright U-shaped suprarostrals in the other three species. This is due to a dorsomedial fusion of the central corpora *vs* ventromedial fusion of the suprarostrals.

Ossification sequences do not vary significantly within a single species, but considerable interspecific variations are observed between the four foam nesters. Differences in the ossification sequence of the cranial bony elements in particular are seen towards the end of metamorphosis. For all four species, the parasphenoid, frontoparietals and exoccipitals are the first bones to ossify, coinciding with the other observations of metamorphic cranial ossification [26]. Paired prootics are the fourth bony elements to form in *Taruga* and *Polypedates;* septomaxilla, maxilla, premaxilla, nasals, dentaries and angulosplenials follow next, showing slight variations in the order of their initial appearance (S1–S4 Tables). Our results show that the major modifications of the chondrocrania occur early in *Taruga* (stages 40–41) when compared with *Polypedates* (stage 42). However, interpretation of metamorphic acceleration or delay is dependent on the ancestral ossification sequence, which cannot be inferred from the existing data. In anurans, it has been recorded that premaxilla, maxilla, septomaxilla and nasals ossify after the underlying nasal cartilages are formed [43], which is also true for these four species. Similarly, the squamosals appear before the metamorphic remodeling of the palatoquadrate in all four species; however, *T*. *eques* shows a comparatively slow ossification rate of this bone. However the order of the appearance of the bones, vomer, pterygoid, quadratojugal, palatine, sphenethmoid, and columella vary considerably among the studied four species (S1–S4 Tables). Ossification of the maxillary arcade commences at the same time in all four species, where maxillae ossify first; however, premaxillae show a slow ossification rate in *P*. *cruciger* and *P*. *maculatus* compared to *T*. *eques* and *T*. *longinasus*.

Regardless the differences in the ossification sequences, the four species studied are similar in the relative timing of the skeletal units; the very first bones are formed in the cranium, followed by the axial skeleton, and next, with a clear delay, the ossification of the forelimbs and hind limbs progress.

Forelimb and hind limb start ossifying simultaneously in all species except in *T*. *longinasus*, where ossification of the forelimbs is initiated first. The osteogensis of the limbs begins only after the I–VIII neural arches are formed. In all four species, the postcranial skeleton is well developed prior to the completion of the ossification of the cranium. Interestingly, tarsals and carpals are only ossified in *Polypedates maculatus* by stage 46, the species with the widest distribution (even across India), whereas in the other three species, these bones were ossified only in adults.

The adult osteology of the two genera also shows several conspicuous differences, helping outline the generic-level boundaries using osteological characters; some of these characters were also used by Liem [10] to define family and generic-level boundaries of rhacophorids. Furthermore, Meegaskumbura *et al*. [14] highlighted some cranial morphological characters (e.g., shape of the skull, frontoparietal, pterygoid, and orbit) to distinguish the two genera. In our study, we highlight two additional characters, which have not been discussed before, i.e., the posteriorly extending parieto-squamosal arch (present in *Polypedates vs*. absent in *Taruga*), and structures of the sternum (“metasternum” [10]) and omosternum. The distinguishable, forked, distally dilated sternum of *P*. *cruciger* is conspicuous among the four; the forked base of the omosternum is broadly forked in *Polypedates*, whereas in *Taruga*, this is not evident.

These results are comparable to published data of the direct developing *Pseudophilautus silus* [17]―a related lineage of *Taruga* and *Polypedates*. The order of cranial bone formation in *Pseudophiluauts silus* is similar to the patterns found in metamorphic anurans rather than the unique sequences found in other well-studied direct developers (e.g., *Eleutherodactlyus coqui* [44]). Direct development removes the need of larval specializations, which can permit developmental repatterning [44]. However, in *P*. *silus* most of the larval specific characters are significantly reduced rather than entirely lost.

Among the deviated characteristics seen in *P*. *silus*, the jaw suspension exhibits the greatest departure from the typical tadpole morphology in its modifications of the palatoquadrate cartilage [17]; the palatoquadrate is present as an initial thin horizontal cartilage (later orienting in a vertical position), where the posterior end of the palatoquadrate is not connected to the neurocranium via ascending and otic processes. Both processes are present in *Taruga* and *Polypedates*.

Initiation of bone formation occurs prior to hatching in this direct-developing species, unlike the foam nesters described here, where the ossification begins after hatching. The formation of the jaw bones (maxilla, premaxilla, dentary, angulosplenial) is accelerated in *P*. *silus* [17], possibly facilitating jaw-usage for active feeding in newly hatched froglets [45]. Vomer, quadratojugal and palatine are absent in hatchlings of *P*. *silus* but are present in adults [17]. However, these three bones are observed by stage 46 in *Taruga* and *Polypedates*. These conspicuous variations between the direct developers and foam nesters, which have been studied so far, indicate the extent of developmental changes associated with these life histories despite sharing a common gel-nesting (GN) ancestor [15].

Characters such as the number of bones present at the end of metamorphosis, ossification sequence and adult cranial morphology are of systematic value, as they tend to consistently vary between species. Our study highlights the variation of these developmental features as they are analyzed in a phylogenetic context.

## Supporting Information

S1 Table Presence/absence (indicated as 1/0 respectively) of cranial and postcranial bones of *Polypedates cruciger* in different developmental stages (stages 25–46). (PDF)

S2 Table Presence/absence (indicated as 1/0 respectively) of cranial and postcranial bones of *Polypedates maculatus* in different developmental stages (stages 25–46). (PDF)

S3 Table Presence/absence (indicated as 1/0 respectively) of cranial and postcranial bones of *Taruga eques* in different developmental stages (stages 25–46). (PDF)

S4 Table Presence/absence (indicated as 1/0 respectively) of cranial and postcranial bones of *Taruga longinasus* in different developmental stages (stages 25–46). (PDF)

S5 Table Taxa included, along with voucher numbers, location and GenBank accession numbers. (PDF)

S6 Table Pairwise divergences between taxa and tadpole gene sequences. (XLSX)

S7 Table Comparison of the ossification sequences of the cranial bones among *Polypedates cruciger*, *P*. *maculatus*, *Taruga eques* and *T*. *longinasus*. (XLSX)

S8 Table Comparison of the ossification sequences of the axial skeletal bones among *Polypedates cruciger*, *P*. *maculatus*, *Taruga eques* and *T*. *longinasus*. (XLSX)

S9 Table Comparison of the ossification sequences of the forelimb and pectoral girdle bones among *Polypedates cruciger*, *P*. *maculatus*, *Taruga eques* and *T*. *longinasus*. (XLSX)

S10 Table Comparison of the ossification sequences of the hind limb and pelvic girdle bones among *Polypedates cruciger*, *P*. *maculatus*, *Taruga eques* and *T*. *longinasus*. (XLSX)

S11 Table Character matrix coded for *Polypedates cruciger*, *P*. *maculatus*, *Taruga eques* and *T*. *longinasus* following the characters recognized by Scott (2005). (XLSX)

## References

[1] Wilkinson JA, Drewes RC. 2000 Character assessment, genus level boundaries, and phylogenetic analyses of the family Rhacophoridae: a review and present day status. Contemporary Herpetology 2:1–24.

[2] Meegaskumbura M, Bossuyt F, Pethiyagoda R, Manamendra-Arachchi K, Bahir M, Milinkovitch M, Schneider CJ. 2002 Sri Lanka: An amphibian hot spot. Science 298:379 10.1126/science.298.5592.379 12376694

[3] Wilkinson JA, Drewes RC, Tatum OL. 2002 A molecular phylogenetic analysis of the family Rhacophoridae with an emphasis on the Asian and African genera. Molecular Phylogenetics and Evolution 24:265–273. 12144761 10.1016/s1055-7903(02)00212-9

[4] Li J, Che J, Bain RH, Zhao E, Zhang Y. 2008 Molecular phylogeny of Rhacophoridae (Anura): a framework of taxonomic reassignment of species within the genera *Aquixalus*, *Chiromantis*, *Rhacophorus* and *Philautus*. Molecular Phylogenetics and Evolution 48:302–312. 10.1016/j.ympev.2008.03.023 18442928

[5] Li J, Che J, Murphy RW, Zhao H, Zhao E, Rao D, Zhang Y. 2009 New insights to the molecular phylogenetics and generic assessment in the Rhacophoridae (Amphibia: Anura) based on five nuclear and three mitochondrial genes, with comments on the evolution of reproduction. Molecular Phylogenetics and Evolution 53:509–522. 10.1016/j.ympev.2009.06.023 19616637

[6] Li J, Li Y, Klaus S, Rao D, Hillis DM, Zhang Y. 2013 Diversification of rhacophorid frogs provides evidence for accelerated faunal exchange between India and Eurasia during the Oligocene. Proceedings of the National Academy of Sciences of the United States of America 110:3441–3446. 10.1073/pnas.1300881110 23401521 PMC3587228

[7] Yu G, Rao D, Yang J, Zang M. 2008 Phylogenetic relationships among Rhacophorinae (Rhacophoridae, Anura, Amphibia), with an emphasis on the Chinese species. Zoological Journal of the Linnean Society 153:733–749.

[8] Yu G, Rao D, Yang J, Zang M. 2009 Re-examination of the phylogeny of Rhacophoridae (Anura) based on mitochondrial and nuclear DNA. Molecular Phylogenetics and Evolution 50:571–579. 10.1016/j.ympev.2008.11.023 19100849

[9] AmphibiaWeb. 2016 AmphibiaWeb: information on amphibian biology and conservation, University of California, Berkeley, California Available at http://www.amphibiaweb.org accessed on 31st January 2015.

[10] Liem SS. 1970 The morphology, systematics, and evolution of the Old World treefrogs (Rhacophoridae and Hyperoliidae). Fieldiana Zoology 57:1–145.

[11] Biju SD, Bossuyt F. 2009 Systematics and phylogeny of *Philautus* Gistel, 1848 (Anura, Rhacophoridae) in the Western Ghats of India, with descriptions of 12 new species. Zoological Journal of the Linnean Society 155:374–444.

[12] Biju SD, Shouche Y, Dubois A, Dutta SK, Bossuyt F. 2010 A ground-dwelling rhacophorid frog from the highest mountain peak of the Western Ghats of India. Current Science 98:1119–1125.

[13] Biju SD, Senevirathne G, Garg S, Mahony S, Kamei RG, Thomas A, et al 2016 *Frankixalus*, a New Rhacophorid Genus of Tree Hole Breeding Frogs with Oophagous Tadpoles. PLoS ONE 11(1):e0145727 10.1371/journal.pone.0145727 26790105 PMC4720377

[14] Meegaskumbura M, Meegaskumbura S, Bowatte G, Manamendra-Arachchi K, Pethiyagoda R, Hanken J, Schneider CJ. 2010 *Taruga* (Anura: Rhacophoridae), a new genus of foam nesting tree frogs endemic to Sri Lanka. Ceylon Journal of Science (Biological Sciences) 39:75–94.

[15] Meegaskumbura M, Senevirathne G, Biju SD, Garg S, Meegaskumbura S, Pethiyagoda R, et al 2015 Patterns of reproductive-mode evolution in Old World tree frogs (Anura, Rhacophoridae). Zoologica Scripta 44(5):509–522.

[16] Abraham RK, Pyron RA, Ansil BR, Zachariah A, Zachariah A. 2013 Two novel genera and one new species of treefrog (Anura: Rhacophoridae) highlight cryptic diversity in the Western Ghats of India. Zootaxa 3640:177–189. 26000411 10.11646/zootaxa.3640.2.3

[17] Kerney R, Meegaskumbura M, Manamendra-Arachchi K, Hanken J. 2007 Cranial ontogeny in *Philautus silus* (Anura: Ranidae: Rhacophorinae) reveals few similarities with other direct-developing anurans. Journal of Morphology 268:715–725. 10.1002/jmor.10545 17538972

[18] Orton GL. 1953 The systematics of vertebrate larvae. Systematic Zoology 2:63–75.

[19] Orton GL. 1957 The bearing of larval evolution on some problems in frog classification. Systematic Zoology 6:79–86.

[20] Ahl E. 1931. Anura III, Polypedatidae. In Das Tierreich. De Gruyter, Berlin and Leipzig. xvi +477 pp.

[21] Hoffman AC.1932 Researches relating to the validity of the south African Polypedatidae (Rhacophoridae) as an autonomous family of the Anura. South African Journal of Sciences 29:562–583.

[22] Laurent R. 1951 Sur la necessite de supprimer la famille des Rhacophoridae mais de creer celle des Hyperoliidae. Revue de Zoologie et de Botanique Africaines 45:116–122.

[23] Gosner KL. 1960 A simplified table for staging anuran embryos and larvae with notes on identification. Herpetologica 16:183–190.

[24] Taylor WR, Van Dyke GC. 1985 Revised procedures for staining and clearing small fishes and other vertebrates for bone and cartilage study. Cybium 9:107–119.

[25] Cannatella D. Architecture: cranial and axial musculoskeleton In: McDiarmid RW, Altig R, editors. Tadpoles: The Biology of Anuran Larvae. Chicago: University of Chicago Press; 1999 pp. 52–91.

[26] Hanken J, Hall BK. 1988 Skull development during anuran metamorphosis: I. Early development of the first three bones to form–the exoccipital, parasphenoid, and frontoparietal. Journal of Morphology 195:247–256. 10.1002/jmor.1051950303 3379643

[27] Trueb L. Bones, frogs, and evolution In: Vial JL, editors. Evolutionary Biology of the Anurans, Contemporary Research on Major Problems. Columbia, MO: University of Missouri Press; 1973 pp. 65–132.

[28] Duellman WE, Trueb L. Biology of the Amphibians. New York: McGraw-Hill; 1986.

[29] Pugener LA, Maglia AM. 1997 Osteology and skeletal development of *Discoglossus sardus* (Anura: Discoglossidae). Journal of Morphology 233:267–286. 10.1002/(SICI)1097-4687(199709)233:3<267::AID-JMOR6>3.0.CO;2-0 9259125

[30] Pugener LA, Maglia AM. 2009 Skeletal morphogenesis of the vertebral column of the miniature hylid frog *Acris crepitans*, with comments on anomalies. Journal of Morphology 270:52–69. 10.1002/jmor.10665 18946872

[31] Pugener A, Maglia AM, Trueb L. 2003 Revisiting the contribution of larval characters to an analysis of phylogenetic relationships of basal anurans. Zoological Journal of Linnean Society 139:129–155.

[32] Maglia AM, Pugener LA, Mueller JM. 2007 Skeletal morphology and adult ontogeny of *Acris crepitans* (Anura: Hylidae): A case of miniaturization in frogs. Journal of Morphology 268:193–282. 10.1002/jmor.10508 17278133

[33] Sambrook J, Fritschi EF, Maniatis T. Molecular cloning: a laboratory manual, New York: Cold Spring Harbor Laboratory Press; 1989.

[34] Palumbi SR. Nucleic acids II: the polymerase chain reaction In: Hillis DM, Moritz C, Mable BK, editors. Molecular Systematics. Sunderland, Sinauer Associates; 1996 pp 205–248.

[35] Tamura K, Peterson D, Peterson N, Stecher G, Nei M, Kumar S. 2011 MEGA 5: Molecular Evolutionary Genetics analysis using Maximum Likelihood, evolutionary distance and Maximum Parsimony methods. Molecular Biology and Evolution 28:2731–2739. 10.1093/molbev/msr121 21546353 PMC3203626

[36] Swofford DL. 2002 PAUP, Phylogenetic Analysis using Parsimony (and other methods), v. 4b10 Sunderland: Sinauer Associates.

[37] Guindon S, Gascuel O. 2003 A simple, fast and accurate method to estimate large phylogenies by maximum-likelihood. Systematic Biology 52:696–704. 14530136 10.1080/10635150390235520

[38] Scott E. 2005 A phylogeny of ranid frogs (Anura: Ranoidea:Ranidae), based on a simultaneous analysis of morphological and molecular data. Cladistics 21:507–574. 10.1111/j.1096-0031.2005.00079.x 34892951

[39] Ridewood WG. 1898 On the larval hyobranchial skeleton of the anurous batrachians. Journal of Linnean Society London 26:474–486.

[40] Okutomi K. 1937 Die Entwicklung des Chondrocranium von *Polypedetes buergeri* schlegelii. Zeitschrift fiir Anatomic und Entwicklungsgeschichte 107:28–64.

[41] Ramaswami LS. 1938 Connexions of the pterygoquadrate in the tadpole of *Philautus variabilis* (Anura). Nature 192:577.

[42] Ramaswami LS. 1956 ‘Frontoparietal’ bone in anura. Current Science (Bangalore) 25:19–20.

[43] Haas A, Richards SJ. 1998 Correlations of cranial morphology, ecology, and evolution in Australian suctorial tadpoles of the genera *Litoria* and *Nyctimystes* (Amphibia: Anura: Hylidae: Pelodryadinae). Journal of Morphology 238(2):109–142. 10.1002/(SICI)1097-4687(199811)238:2<109::AID-JMOR1>3.0.CO;2-# 9796529

[44] Hanken J, Klymkowsky MW, Summers CH, Seufert DW, Ingebrigtsen N. 1992 Cranial ontogeny in the direct-developing frog, *Eleutherodactylus coqui* (Anura: Leptodactylidae), analyzed using whole mount immunohistochemistry. Journal of Morphology 211:95–118. 10.1002/jmor.1052110111 1371162

[45] Yeh J. 2002 The evolution of development: Two portraits of skull ossification in pipoid frogs. Evolution 56:2484–2498. 12583588 10.1111/j.0014-3820.2002.tb00173.x

