# Supplementary material for: Comparative Postembryonic Skeletal Ontogeny in Two Sister Lineages of Old World Tree Frogs (Rhacophoridae: Taruga, Polypedates)
Source: PLoS One. 2017 Jan 6;12(1):e0167939. doi: 10.1371/journal.pone.0167939 (PMC5218391; doi:10.1371/journal.pone.0167939)
Supplement: S2 Table — (PDF) [file pone.0167939.s002.pdf]

	25	26	27	28	29	30	31	32	32	32	33	34	34	34	35	35	35	36	37	38	39	40
Skull Bones																						
Exoccipital	0	0	0	1	1	1	1	1	1	1	1	1	1	1	1	1	1	1	1	1	1	1
Parasphenoid	0	0	1	1	1	1	1	1	1	1	1	1	1	1	1	1	1	1	1	1	1	1
Frontoparietal	0	0	0	1	1	1	1	1	1	1	1	1	1	1	1	1	1	1	1	1	1	1
Prootic	0	0	0	0	0	0	0	1	1	1	1	1	1	1	1	1	1	1	1	1	1	
Septomaxill	0	0	0	0	0	0	0	0	0	0	0	0	0	0	0	0	0	0	0	0	0	0
Premaxilla	0	0	0	0	0	0	0	0	0	0	0	0	0	0	0	0	0	0	0	0	0	0
Maxilla	0	0	0	0	0	0	0	0	0	0	0	0	0	0	0	0	0	0	0	0	0	0
Nasal	0	0	0	0	0	0	0	0	0	0	0	0	0	0	0	0	0	0	0	0	0	0
Squamosal	0	0	0	0	0	0	0	0	0	0	0	0	0	0	0	0	0	0	0	0	0	0
Angulosplenia	0	0	0	0	0	0	0	0	0	0	0	0	0	0	0	0	0	0	0	0	0	0
Vomer	0	0	0	0	0	0	0	0	0	0	0	0	0	0	0	0	0	0	0	0	0	0
Dentary	0	0	0	0	0	0	0	0	0	0	0	0	0	0	0	0	0	0	0	0	0	0
Pterygoid	0	0	0	0	0	0	0	0	0	0	0	0	0	0	0	0	0	0	0	0	0	0
Quadratojugal	0	0	0	0	0	0	0	0	0	0	0	0	0	0	0	0	0	0	0	0	0	0
Columella	0	0	0	0	0	0	0	0	0	0	0	0	0	0	0	0	0	0	0	0	0	0
Mentomeckelian	0	0	0	0	0	0	0	0	0	0	0	0	0	0	0	0	0	0	0	0	0	0
Palatine	0	0	0	0	0	0	0	0	0	0	0	0	0	0	0	0	0	0	0	0	0	0
Teeth	0	0	0	0	0	0	0	0	0	0	0	0	0	0	0	0	0	0	0	0	0	0
Sphenethmiod	0	0	0	0	0	0	0	0	0	0	0	0	0	0	0	0	0	0	0	0	0	0
**Hydrobeanchial**																						
Hyoid	0	0	0	0	0	0	0	0	0	0	0	0	0	0	0	0	0	0	0	0	0	0
**Axial**																						
Neural arch 1	0	0	1	1	1	1	1	1	1	1	1	1	1	1	1	1	1	1	1	1	1	1
Neural arch 2	0	0	1	1	1	1	1	1	1	1	1	1	1	1	1	1	1	1	1	1	1	1
Neural arch 3	0	0	1	1	1	1	1	1	1	1	1	1	1	1	1	1	1	1	1	1	1	1
Neural arch 4	0	0	1	1	1	1	1	1	1	1	1	1	1	1	1	1	1	1	1	1	1	1
Neural arch 5	0	0	1	1	1	1	1	1	1	1	1	1	1	1	1	1	1	1	1	1	1	1
Neural arch 6	0	0	0	1	1	1	1	1	1	1	1	1	1	1	1	1	1	1	1	1	1	1
Neural arch 7	0	0	0	1	1	1	1	1	1	1	1	1	1	1	1	1	1	1	1	1	1	1
Neural arch 8	0	0	0	0	1	1	1	1	1	1	1	1	1		1	1	1	1	1	1	1	1
sacral	0	0	0	0	0	0	0	0	0	0	0	0	0	0	0	0	0	0	1	1	1	1
Centra1	0	0	0	0	1	1	1	1	1	1	1	1	1	1	1	1	1	1	1	1	1	1
Centra2	0	0	0	0	0	1	1	1	1	1	1	1	1	1	1	1	1	1	1	1	1	1
Centra3	0	0	0	0	0	1	1	1	1	1	1	1	1	1	1	1	1	1	1	1	1	1
Centra4	0	0	0	0	0	1	1	1	1	1	1	1	1	1	1	1	1	1	1	1	1	1
Centra5	0	0	0	0	0	0	0	0	1	1	1	1	1	1	1	1	1	1	1	1	1	1
Centra6	0	0	0	0	0	0	0	0	0	0	1	1	1	1	1	1	1	1	1	1	1	1
Centra7	0	0	0	0	0	0	0	0	0	0	0	1	1	1	1	1	1	1	1	1	1	1
Centra8	0	0	0	0	0	0	0	0	0	0	0	1	1	1	1	1	1	1	1	1	1	1
sacral diapophysis	0	0	0	0	0	0	0	0	0	0	0	0	0	0	0	0	0	0	0	1	1	1
Hypochord	0	0	0	0	0	0	0	0	0	0	0	0	0	0	0	0	0	0	0	0	0	0

Coccyx	0	0	0	0	0	0	0	0	0	0	0	0	0	0	0	0	0	0	0	0	0	1
Urostyle	0	0	0	0	0	0	0	0	0	0	0	0	0	0	0	0	0	0	0	0	0	1
**Fore limbs**																						
Scapula	0	0	0	0	0	0	0	0	0	0	0	0	0	0	0	0	1	1	1	1	1	1
Humerus	0	0	0	0	0	0	0	0	0	0	0	0	0	0	1	1	1	1	1	1	1	1
Ulna	0	0	0	0	0	0	0	0	0	0	0	0	0	0	0	1	1	1	1	1	1	1
	25	26	27	28	29	30	31	32	32	32	33	34	34	34	35	35	35	36	37	38	39	40
Radius	0	0	0	0	0	0	0	0	0	0	0	0	0	0	0	1	1	1	1	1	1	1
Cleithrum	0	0	0	0	0	0	0	0	0	0	0	0	0	0	0	0	0	0	1	1	1	1
Clavicle	0	0	0	0	0	0	0	0	0	0	0	0	0	0	0	0	0	0	0	0	0	0
Coracoid	0	0	0	0	0	0	0	0	0	0	0	0	0	0	0	0	0	0	0	0	0	0
Metacarpals	0	0	0	0	0	0	0	0	0	0	0	0	0	0	0	0	0	0	0	0	0	0
Phalanges digits	0	0	0	0	0	0	0	0	0	0	0	0	0	0	0	0	0	0	0	0	0	0
Sternum	0	0	0	0	0	0	0	0	0	0	0	0	0	0	0	0	0	0	0	0	0	0
Osmosternum	0	0	0	0	0	0	0	0	0	0	0	0	0	0	0	0	0	0	0	0	0	0
**Hind Limb**																						
Femur	0	0	0	0	0	0	0	0	0	0	0	0	0	0	1	1	1	1	1	1	1	1
Tibia	0	0	0	0	0	0	0	0	0	0	0	0	0	0	0	1	1	1	1	1	1	1
Fibula	0	0	0	0	0	0	0	0	0	0	0	0	0	0	0	1	1	1	1	1	1	1
Fibulare	0	0	0	0	0	0	0	0	0	0	0	0	0	0	0	0	0	0	0	1	1	1
Tibiale	0	0	0	0	0	0	0	0	0	0	0	0	0	0	0	0	0	0	0	1	1	1
Metatarsalia	0	0	0	0	0	0	0	0	0	0	0	0	0	0	0	0	0	0	0	1	1	1
Ilium	0	0	0	0	0	0	0	0	0	0	0	0	0	0	0	0	0	0	0	1	1	1
Ischium	0	0	0	0	0	0	0	0	0	0	0	0	0	0	0	0	0	0	0	0	0	0
phalange digits	0	0	0	0	0	0	0	0	0	0	0									1	1	1
trans processes 1	0	0	0	0	0	0	0	0	0	0	0	0	0	0	0	0	0	0	0	0	0	0
trans processes 2	0	0	0	0	1	1	1	1	1	1	1	1	1	1	1	1	1	1	1	1	1	1
trans processes 3	0	0	0	0	0	0	1	1	1	1	1	1	1	1	1	1	1	1	1	1	1	1
trans processes 4	0	0	0	0	0	0	0	0	0	0	0	0	0	0	0	0	0	0	0	1	1	1
trans processes 5	0	0	0	0	0	0	0	0	0	0	0	0	0	0	0	0	0	0	0	0	0	0
trans processes 6	0	0	0	0	0	0	0	0	0	0	0	0	0	0	0	0	0	0	0	0	0	0
trans processes 7	0	0	0	0	0	0	0	0	0	0	0	0	0	0	0	0	0	0	0	0	0	0
trans processes 8	0	0	0	0	0	0	0	0	0	0	0	0	0	0	0	0	0	0	0	0	0	0
carpels	0	0	0	0	0	0	0	0	0	0	0	0	0	0	0	0	0	0	0	0	0	0
Tarsals	0	0	0	0	0	0	0	0	0	0	0	0	0	0	0	0	0	0	0	0	0	0

	41	42	43	44	45	46
Skull Bones						
Exoccipital	1	1	1	1	1	1
Parasphenoid	1	1	1	1	1	1
Frontoparietal	1	1	1	1	1	1
Prootic	1	1	1	1	1	1
Septomaxill	0	1	1	1	1	1
Premaxilla	0	1	1	1	1	1
Maxilla	0	1	1	1	1	1
Nasal	0	1	1	1	1	1
Squamosal	0	0	1	1	1	1
Angulosplenia	0	1	1	1	1	1
Vomer	0	0	1	1	1	1
Dentary	0	1	1	1	1	1
Pterygoid	0	1	1	1	1	1
Quadratojugal	0	1	1	1	1	1
Columella	0	0	1	1	1	1
Mentomeckelian	0	0	0	0	0	1
Palatine	0	0	1	1	1	1
Teeth	0	0	1	1	1	1
Sphenethmiod	0	0	1	1	1	1
**Hydrobeanchial**						
Hyoid	0	0	0	0	0	0
**Axial**						
Neural arch 1	1	1	1	1	1	1
Neural arch 2	1	1	1	1	1	1
Neural arch 3	1	1	1	1	1	1
Neural arch 4	1	1	1	1	1	1
Neural arch 5	1	1	1	1	1	1
Neural arch 6	1	1	1	1	1	1
Neural arch 7	1	1	1	1	1	1
Neural arch 8	1	1	1	1	1	1
sacral	1	1	1	1	1	1
Centra1	1	1	1	1	1	1
Centra2	1	1	1	1	1	1
Centra3	1	1	1	1	1	1
Centra4	1	1	1	1	1	1
Centra5	1	1	1	1	1	1
Centra6	1	1	1	1	1	1
Centra7	1	1	1	1	1	1
Centra8	1	1	1	1	1	1
sacral diapophysis	1	1	1	1	1	1
Hypochord	0	0	0	1	1	1

Coccyx	0	0	1	1	1	1
Urostyle	1	1	1	1	1	1
**Fore limbs**						
Scapula	1	1	1	1	1	1
Humerus	1	1	1	1	1	1
Ulna	1	1	1	1	1	1
	41	42	43	44	45	46
Radius	1	1	1	1	1	1
Cleithrum	1	1	1	1	1	1
Clavicle	1	1	1	1	1	1
Coracoid	1	1	1	1	1	1
Metacarpals	1	1	1	1	1	1
Phalanges digits	1	1	1	1	1	1
Sternum	0	0	0	0	0	1
Osmosternum	0	0	0	0	0	1
**Hind Limb**						1
Femur	1	1	1	1	1	1
Tibia	1	1	1	1	1	1
Fibula	1	1	1	1	1	1
Fibulare	1	1	1	1	1	1
Tibiale	1	1	1	1	1	1
Metatarsalia	1	1	1	1	1	1
Ilium	1	1	1	1	1	1
Ischium	0	0	1	1	1	1
phalange digits	1	1	1	1	1	1
trans processes 1	0	0	0	0	0	0
trans processes 2	1	1	1	1	1	1
trans processes 3	1	1	1	1	1	1
trans processes 4	1	1	1	1	1	1
trans processes 5	1	1	1	1	1	1
trans processes 6	1	1	1	1	1	1
trans processes 7	1	1	1	1	1	1
trans processes 8	1	1	1	1	1	1
carpels	0	0	0	0	0	1
Tarsals	0	0	0	0	0	1
