# Supplementary material for: Comparative Postembryonic Skeletal Ontogeny in Two Sister Lineages of Old World Tree Frogs (Rhacophoridae: Taruga, Polypedates)
Source: PLoS One. 2017 Jan 6;12(1):e0167939. doi: 10.1371/journal.pone.0167939 (PMC5218391; doi:10.1371/journal.pone.0167939)
Supplement: S3 Table — (PDF) [file pone.0167939.s003.pdf]

Skull Bones	26	26	26	26	26	27	27	27	27	27	28	28	28	28	29	29	29	30	31	31	31	31	32
Exoccipital	0	0	0	0	0	1	1	1	1	1	1	1	1	1	1	1	1	1	1	1	1	1	1
Parasphenoid	0	0	0	1	1	1	1	1	1	1	1	1	1	1	1	1	1	1	1	1	1	1	1
Frontoparietal	0	0	0	0	0	0	0	1	1	1	1	1	1	1	1	1	1	1	1	1	1	1	1
Prootic	0	0	0	0	0	0	0	0	0	0	0	0	0	0	0	0	0	0	0	0	0	0	0
Septomaxill	0	0	0	0	0	0	0	0	0	0	0	0	0	0	0	0	0	0	0	0	0	0	0
Premaxilla	0	0	0	0	0	0	0	0	0	0	0	0	0	0	0	0	0	0	0	0	0	0	0
Maxilla	0	0	0	0	0	0	0	0	0	0	0	0	0	0	0	0	0	0	0	0	0	0	0
Nasal	0	0	0	0	0	0	0	0	0	0	0	0	0	0	0	0	0	0	0	0	0	0	0
Squamosal	0	0	0	0	0	0	0	0	0	0	0	0	0	0	0	0	0	0	0	0	0	0	0
Angulosplenia	0	0	0	0	0	0	0	0	0	0	0	0	0	0	0	0	0	0	0	0	0	0	0
Vomer	0	0	0	0	0	0	0	0	0	0	0	0	0	0	0	0	0	0	0	0	0	0	0
Dentary	0	0	0	0	0	0	0	0	0	0	0	0	0	0	0	0	0	0	0	0	0	0	0
Pterygoid	0	0	0	0	0	0	0	0	0	0	0	0	0	0	0	0	0	0	0	0	0	0	0
Quadratojugal	0	0	0	0	0	0	0	0	0	0	0	0	0	0	0	0	0	0	0	0	0	0	0
Columella	0	0	0	0	0	0	0	0	0	0	0	0	0	0	0	0	0	0	0	0	0	0	0
Mentomeckelian	0	0	0	0	0	0	0	0	0	0	0	0	0	0	0	0	0	0	0	0	0	0	0
Palatine	0	0	0	0	0	0	0	0	0	0	0	0	0	0	0	0	0	0	0	0	0	0	0
Teeth	0	0	0	0	0	0	0	0	0	0	0	0	0	0	0	0	0	0	0	0	0	0	0
Sphenethmiod	0	0	0	0	0	0	0	0	0	0	0	0	0	0	0	0	0	0	0	0	0	0	0
**Hyobranchial**																							
Hyoid	0	0	0	0	0	0	0	0	0	0	0	0	0	0	0	0	0	0	0	0	0	0	0
**Axial**																							
Neural arch 1	0	0	0	1	1	1	1	1	1	1	1	1	1	1	1	1	1	1	1	1	1	1	1
Neural arch 2	0	0	0	1	1	1	1	1	1	1	1	1	1	1	1	1	1	1	1	1	1	1	1
Neural arch 3	0	0	0	1	1	1	1	1	1	1	1	1	1	1	1	1	1	1	1	1	1	1	1
Neural arch 4	0	0	0	1	1	1	1	1	1	1	1	1	1	1	1	1	1	1	1	1	1	1	1
Neural arch 5	0	0	0	0	1	1	1	1	1	1	1	1	1	1	1	1	1	1	1	1	1	1	1
Neural arch 6	0	0	0	0	1	1	1	1	1	1	1	1	1	1	1	1	1	1	1	1	1	1	1
Neural arch 7	0	0	0	0	1	1	1	1	1	1	1	1	1	1	1	1	1	1	1	1	1	1	1
Neural arch 8	0	0	0	0	0	1	1	1	1	1	1	1	1	1	1	1	1	1	1	1	1	1	1
sacral	0	0	0	0	0	0	0	0	0	0	0	0	0	0	1	1	1	1	1	1	1	1	1
Centra1	0	0	0	1	1	1	1	1	1	1	1	1	1	1	1	1	1	1	1	1	1	1	1
Centra2	0	0	0	1	1	1	1	1	1	1	1	1	1	1	1	1	1	1	1	1	1	1	1
Centra3	0	0	0	1	1	1	1	1	1	1	1	1	1	1	1	1	1	1	1	1	1	1	1
Centra4	0	0	0	0	1	1	1	1	1	1	1	1	1	1	1	1	1	1	1	1	1	1	1
Centra5	0	0	0	0	1	1	1	1	1	1	1	1	1	1	1	1	1	1	1	1	1	1	1
Centra6	0	0	0	0	1	1	1	1	1	1	1	1	1	1	1	1	1	1	1	1	1	1	1
Centra7	0	0	0	0	1	1	1	1	1	1	1	1	1	1	1	1	1	1	1	1	1	1	1
Centra8	0	0	0	0	0	1	1	1	1	1	1	1	1	1	1	1	1	1	1	1	1	1	1
sacral diapophysis	0	0	0	0	0	0	0	0	0	0	1	1	1	1	1	1	1	1	1	1	1	1	1
Hypochord	0	0	0	0	0	0	0	0	0	0	1	1	1	1	1	1	1	1	1	1	1	1	1
Coccyx	0	0	0	0	0	0	0	0	0	0	1	1	1	1	1	1	1	1	1	1	1	1	1
UROSTYLE	0	0	0	0	0	0	0	0	0	0	0	0	0	0	0	0	0	0	0	0	0	0	0

	26	26	26	26	26	27	27	27	27	27	28	28	28	28	29	29	29	30	31	31	31	31	32
**Fore limbs**																							
Scapula	0	0	0	0	0	0	0	0	0	0	0	0	0	0	0	0	0	0	0	0	0	0	0
Humerus	0	0	0	0	0	0	0	0	0	0	0	0	0	0	0	0	0	0	0	0	0	0	0
Ulna	0	0	0	0	0	0	0	0	0	0	0	0	0	0	0	0	0	0	0	0	0	0	0
Radius	0	0	0	0	0	0	0	0	0	0	0	0	0	0	0	0	0	0	0	0	0	0	0
Cleithrum	0	0	0	0	0	0	0	0	0	0	0	0	0	0	0	0	0	0	0	0	0	0	0
Clavicle	0	0	0	0	0	0	0	0	0	0	0	0	0	0	0	0	0	0	0	0	0	0	0
Coracoid	0	0	0	0	0	0	0	0	0	0	0	0	0	0	0	0	0	0	0	0	0	0	0
Metacarpals	0	0	0	0	0	0	0	0	0	0	0	0	0	0	0	0	0	0	0	0	0	0	0
Phalanges digits	0	0	0	0	0	0	0	0	0	0	0	0	0	0	0	0	0	0	0	0	0	0	0
Sternum	0	0	0	0	0	0	0	0	0	0	0	0	0	0	0	0	0	0	0	0	0	0	0
Osmosternum	0	0	0	0	0	0	0	0	0	0	0	0	0	0	0	0	0	0	0	0	0	0	0
**Hind Limb**																							
Femur	0	0	0	0	0	0	0	0	0	0	0	0	0	0	0	0	0	0	0	0	0	0	0
Tibia	0	0	0	0	0	0	0	0	0	0	0	0	0	0	0	0	0	0	0	0	0	0	0
Fibula	0	0	0	0	0	0	0	0	0	0	0	0	0	0	0	0	0	0	0	0	0	0	0
Fibulare	0	0	0	0	0	0	0	0	0	0	0	0	0	0	0	0	0	0	0	0	0	0	0
Tibiale	0	0	0	0	0	0	0	0	0	0	0	0	0	0	0	0	0	0	0	0	0	0	0
Metatarsalia	0	0	0	0	0	0	0	0	0	0	0	0	0	0	0	0	0	0	0	0	0	0	0
Ilium	0	0	0	0	0	0	0	0	0	0	0	0	0	0	0	0	0	0	0	0	0	0	0
Ischium	0	0	0	0	0	0	0	0	0	0	0	0	0	0	0	0	0	0	0	0	0	0	0
trans processes 1	0	0	0	0	0	0	0	0	0	0	0	0	1	1	1	1	1	1	1	1	1	1	1
trans processes 2	0	0	0	0	0	1	1	1	1	1	1	1	1	1	1	1	1	1	1	1	1	1	1
trans processes 3	0	0	0	0	0	1	1	1	1	1	1	1	1	1	1	1	1	1	1	1	1	1	1
trans processes 4	0	0	0	0	0	0	0	0	0	0	0	0	0	0	0	0	0	0	0	0	0	0	0
trans processes 5	0	0	0	0	0	0	0	0	0	0	0	0	0	0	0	0	0	0	0	0	0	0	0
trans processes 6	0	0	0	0	0	0	0	0	0	0	0	0	0	0	0	0	0	0	0	0	0	0	0
trans processes 7	0	0	0	0	0	0	0	0	0	0	0	0	0	0	0	0	0	0	0	0	0	0	0
trans processes 8	0	0	0	0	0	0	0	0	0	0	0	0	0	0	0	0	0	0	0	0	0	0	0

**Skull Bones**	32	32	33	33	33	33	34	34	34	35	35	35	35	36	36	36	36	37	37	37	37	38	38
Exoccipital	1	1	1	1	1	1	1	1	1	1	1	1	1	1	1	1	1	1	1	1	1	1	1
Parasphenoid	1	1	1	1	1	1	1	1	1	1	1	1	1	1	1	1	1	1	1	1	1	1	1
Frontoparietal	1	1	1	1	1	1	1	1	1	1	1	1	1	1	1	1	1	1	1	1	1	1	1
Prootic	0	0	0	0	0	0	1	1	1	1	1	1	1	1	1	1	1	1	1	1	1	1	1
Septomaxill	0	0	0	0	0	0	0	0	0	0	0	0	0	0	0	0	0	0	0	0	0	0	0
Premaxilla	0	0	0	0	0	0	0	0	0	0	0	0	0	0	0	0	0	0	0	0	0	0	0
Maxilla	0	0	0	0	0	0	0	0	0	0	0	0	0	0	0	0	0	0	0	0	0	0	0
Nasal	0	0	0	0	0	0	0	0	0	0	0	0	0	0	0	0	0	0	0	0	0	0	0
Squamosal	0	0	0	0	0	0	0	0	0	0	0	0	0	0	0	0	0	0	0	0	0	0	0
Angulosplenia	0	0	0	0	0	0	0	0	0	0	0	0	0	0	0	0	0	0	0	0	0	0	0
Vomer	0	0	0	0	0	0	0	0	0	0	0	0	0	0	0	0	0	0	0	0	0	0	0
Dentary	0	0	0	0	0	0	0	0	0	0	0	0	0	0	0	0	0	0	0	0	0	0	0
Pterygoid	0	0	0	0	0	0	0	0	0	0	0	0	0	0	0	0	0	0	0	0	0	0	0
Quadratojugal	0	0	0	0	0	0	0	0	0	0	0	0	0	0	0	0	0	0	0	0	0	0	0
Columella	0	0	0	0	0	0	0	0	0	0	0	0	0	0	0	0	0	0	0	0	0	0	0
Mentomeckelian	0	0	0	0	0	0	0	0	0	0	0	0	0	0	0	0	0	0	0	0	0	0	0
Palatine	0	0	0	0	0	0	0	0	0	0	0	0	0	0	0	0	0	0	0	0	0	0	0
Teeth	0	0	0	0	0	0	0	0	0	0	0	0	0	0	0	0	0	0	0	0	0	0	0
Sphenethmiod	0	0	0	0	0	0	0	0	0	0	0	0	0	0	0	0	0	0	0	0	0	0	0

Hyobranchial

Hyoid	0	0	0	0	0	0	0	0	0	0	0	0	0	0	0	0	0	0	0	0	0	0	0

Axial

Neural arch 1	1	1	1	1	1	1	1	1	1	1	1	1	1	1	1	1	1	1	1	1	1	1	1
Neural arch 2	1	1	1	1	1	1	1	1	1	1	1	1	1	1	1	1	1	1	1	1	1	1	1
Neural arch 3	1	1	1	1	1	1	1	1	1	1	1	1	1	1	1	1	1	1	1	1	1	1	1
Neural arch 4	1	1	1	1	1	1	1	1	1	1	1	1	1	1	1	1	1	1	1	1	1	1	1
Neural arch 5	1	1	1	1	1	1	1	1	1	1	1	1	1	1	1	1	1	1	1	1	1	1	1
Neural arch 6	1	1	1	1	1	1	1	1	1	1	1	1	1	1	1	1	1	1	1	1	1	1	1
Neural arch 7	1	1	1	1	1	1	1	1	1	1	1	1	1	1	1	1	1	1	1	1	1	1	1
Neural arch 8	1	1	1	1	1	1	1	1	1	1	1	1	1	1	1	1	1	1	1	1	1	1	1
sacral	1	1	1	1	1	1	1	1	1	1	1	1	1	1	1	1	1	1	1	1	1	1	1
Centra1	1	1	1	1	1	1	1	1	1	1	1	1	1	1	1	1	1	1	1	1	1	1	1
Centra2	1	1	1	1	1	1	1	1	1	1	1	1	1	1	1	1	1	1	1	1	1	1	1
Centra3	1	1	1	1	1	1	1	1	1	1	1	1	1	1	1	1	1	1	1	1	1	1	1
Centra4	1	1	1	1	1	1	1	1	1	1	1	1	1	1	1	1	1	1	1	1	1	1	1
Centra5	1	1	1	1	1	1	1	1	1	1	1	1	1	1	1	1	1	1	1	1	1	1	1
Centra6	1	1	1	1	1	1	1	1	1	1	1	1	1	1	1	1	1	1	1	1	1	1	1
Centra7	1	1	1	1	1	1	1	1	1	1	1	1	1	1	1	1	1	1	1	1	1	1	1
Centra8	1	1	1	1	1	1	1	1	1	1	1	1	1	1	1	1	1	1	1	1	1	1	1
sacral diapophysis	1	1	1	1	1	1	1	1	1	1	1	1	1	1	1	1	1	1	1	1	1	1	1
Hypochord	1	1	1	1	1	1	1	1	1	1	1	1	1	1	1	1	1	1	1	1	1	1	1
Coccyx	1	1	1	1	1	1	1	1	1	1	1	1	1	1	1	1	1	1	1	1	1	1	1
UROSTYLE	0	1	1	0	0	1	1	1	1	1	1	1	1	1	1	1	1	1	1	1	1	1	1

	32	32	33	33	33	33	34	34	34	35	35	35	35	36	36	36	36	37	37	37	37	38	38
**Fore limbs**																							
Scapula	0	0	0	0	0	0	0	0	0	1	1	1	1	1	1	1	1	1	1	1	1	1	1
Humerus	0	0	0	0	0	0	0	0	0	1	1	1	1	1	1	1	1	1	1	1	1	1	1
Ulna	0	0	0	0	0	0	0	0	0	1	1	1	1	1	1	1	1	1	1	1	1	1	1
Radius	0	0	0	0	0	0	0	0	0	1	1	1	1	1	1	1	1	1	1	1	1	1	1
Cleithrum	0	0	0	0	0	0	0	0	0	0	0	*	*	0	0	0	0	1	1	1	1	1	1
Clavicle	0	0	0	0	0	0	0	0	0	0	0	*	*	0	0	0	0	1	1	1	1	1	1
Coracoid	0	0	0	0	0	0	0	0	0	0	0	*	*	0	0	0	0	1	1	1	1	1	1
Metacarpals	0	0	0	0	0	0	0	0	0	0	0	*	*	0	0	0	0	0	0	0	0	0	0
Phalanges digits	0	0	0	0	0	0	0	0	0	0	0	*	*	0	0	0	0	0	0	0	0	0	0
Sternum	0	0	0	0	0	0	0	0	0	0	0	*	*	0	0	0	0	0	0	0	0	0	0
Osmosternum	0	0	0	0	0	0	0	0	0	0	0	*	*	0	0	0	0	0	0	0	0	0	0
**Hind Limb**																							
Femur	0	0	0	0	0	0	0	0	0	1	1	1	1	1	1	1	1	1	1	1	1	1	1
Tibia	0	0	0	0	0	0	0	0	0	1	0	0	*	1	1	1	1	1	1	1	1	1	1
Fibula	0	0	0	0	0	0	0	0	0	1	0	0	*	1	1	1	1	1	1	1	1	1	1
Fibulare	0	0	0	0	0	0	0	0	0	0	0	0	*	0	0	0	0	1	1	1	1	1	1
Tibiale	0	0	0	0	0	0	0	0	0	0	0	0	*	0	0	0	0	1	1	1	1	1	1
Metatarsalia	0	0	0	0	0	0	0	0	0	0	0	0	*	0	0	0	0	0	0	0	0	0	0
Ilium	0	0	0	0	0	0	0	0	0	1	1	1	*	1	1	1	1	1	1	1	1	1	1
Ischium	0	0	0	0	0	0	0	0	0	0	0	0	*	0	0	0	0	0	0	0	0	0	0
trans processes 1	1	1	1	1	1	1	1	1	1	1	1	1	1	1	1	1	1	1	1	1	1	1	1
trans processes 2	1	1	1	1	1	1	1	1	1	1	1	1	1	1	1	1	1	1	1	1	1	1	1
trans processes 3	1	1	1	1	1	1	1	1	1	1	1	1	1	1	1	1	1	1	1	1	1	1	1
trans processes 4	0	0	0	0	0	0	0	0	0	0	0	0	0	1	1	1	1	1	1	1	1	1	1
trans processes 5	0	0	0	0	0	0	0	0	0	0	0	0	0	1	1	1	1	1	1	1	1	1	1
trans processes 6	0	0	0	0	0	0	0	0	0	0	0	0	0	1	1	1	1	1	1	1	1	1	1
trans processes 7	0	0	0	0	0	0	0	0	0	0	0	0	0	1	1	1	1	1	1	1	1	1	1
trans processes 8	0	0	0	0	0	0	0	0	0	0	0	0	0	0	0	0	0	0	1	1	1	1	1

38	39	39	39	**Skull Bones**	40	40	40	40	41	41	41	42	42	43	43	44	45	46
1	1	1	1	Exoccipital	1	1	1	1	1	1	1	1	1	1	1	1	1	1
1	1	1	1	Parasphenoid	1	1	1	1	1	1	1	1	1	1	1	1	1	1
1	1	1	1	Frontoparietal	1	1	1	1	1	1	1	1	1	1	1	1	1	1
1	1	1	1	Prootic	1	1	1	1	1	1	1	1	1	1	1	1	1	1
0	0	0	0	Septomaxill	1	0	1	1	1	1	1	1	1	1	1	1	1	1
0	0	0	0	Premaxilla	1	0	1	1	1	1	1	1	1	1	1	1	1	1
0	0	0	0	Maxilla	0	0	0	0	0	0	0	1	1	1	1	1	1	1
0	0	0	0	Nasal	0	0	0	0	0	0	0	0	0	1	1	1	1	1
0	0	0	0	Squamosal	0	0	0	0	0	0	0	0	0	0	0	1	1	1
0	0	0	0	Angulosplenia	0	0	0	0	0	0	0	0	0	1	1	1	1	1
0	0	0	0	Vomer	0	0	0	0	0	0	0	0	0	0	0	0	0	1
0	0	0	0	Dentary	0	0	0	0	0	0	0	0	0	0	0	0	1	1
0	0	0	0	Pterygoid	0	0	0	0	0	0	0	0	0	0	0	0	1	1
0	0	0	0	Quadratojugal	0	0	0	0	0	0	0	0	0	0	0	0	0	1
0	0	0	0	Columella	0	0	0	0	0	0	0	0	0	0	0	0	1	1
0	0	0	0	Mentomeckelian	0	0	0	0	0	0	0	0	0	0	0	0	1	1
0	0	0	0	Palatine	0	0	0	0	0	0	0	0	0	0	0	0	1	1
0	0	0	0	Teeth	0	0	0	0	0	0	0	0	0	0	0	0	0	0
0	0	0	0	Sphenethmiod	0	0	0	0	0	0	0	0	0	0	0	0	0	0
				**Hyobranchial**														
0	0	0	0	Hyoid	0	0	0	0	0	0	0	0	0	0	0	0	0	1
				**Axial**														
1	1	1	1	Neural arch 1	1	1	1	1	1	1	1	1	1	1	1	1	1	1
1	1	1	1	Neural arch 2	1	1	1	1	1	1	1	1	1	1	1	1	1	1
1	1	1	1	Neural arch 3	1	1	1	1	1	1	1	1	1	1	1	1	1	1
1	1	1	1	Neural arch 4	1	1	1	1	1	1	1	1	1	1	1	1	1	1
1	1	1	1	Neural arch 5	1	1	1	1	1	1	1	1	1	1	1	1	1	1
1	1	1	1	Neural arch 6	1	1	1	1	1	1	1	1	1	1	1	1	1	1
1	1	1	1	Neural arch 7	1	1	1	1	1	1	1	1	1	1	1	1	1	1
1	1	1	1	Neural arch 8	1	1	1	1	1	1	1	1	1	1	1	1	1	1
1	1	1	1	sacral	1	1	1	1	1	1	1	1	1	1	1	1	1	1
1	1	1	1	Centra1	1	1	1	1	1	1	1	1	1	1	1	1	1	1
1	1	1	1	Centra2	1	1	1	1	1	1	1	1	1	1	1	1	1	1
1	1	1	1	Centra3	1	1	1	1	1	1	1	1	1	1	1	1	1	1
1	1	1	1	Centra4	1	1	1	1	1	1	1	1	1	1	1	1	1	1
1	1	1	1	Centra5	1	1	1	1	1	1	1	1	1	1	1	1	1	1
1	1	1	1	Centra6	1	1	1	1	1	1	1	1	1	1	1	1	1	1
1	1	1	1	Centra7	1	1	1	1	1	1	1	1	1	1	1	1	1	1
1	1	1	1	Centra8	1	1	1	1	1	1	1	1	1	1	1	1	1	1
1	1	1	1	sacral diapophysis	1	1	1	1	1	1	1	1	1	1	1	1	1	1
1	1	1	1	Hypochord	1	1	1	1	1	1	1	1	1	1	1	1	1	1
1	1	1	1	Coccyx	1	1	1	1	1	1	1	1	1	1	1	1	1	1
1	1	1	1	UROSTYLE	1	1	1	1	1	1	1	1	1	1	1	1	1	1

38	39	39	39		40	40	40	40	41	41	41	42	42	43	43	44	45	46
				**Fore limbs**														
1	1	1	1	Scapula	1	1	1	1	1	1	1	1	1	1	1	1	1	1
1	1	1	1	Humerus	1	1	1	1	1	1	1	1	1	1	1	1	1	1
1	1	1	1	Ulna	1	1	1	1	1	1	1	1	1	1	1	1	1	1
1	1	1	1	Radius	1	1	1	1	1	1	1	1	1	1	1	1	1	1
1	1	1	1	Cleithrum	1	1	1	1	1	1	1	1	1	1	1	1	1	1
1	1	1	1	Clavicle	1	1	1	1	1	1	1	1	1	1	1	1	1	1
1	1	1	1	Coracoid	1	1	1	1	1	1	1	1	1	1	1	1	1	1
0	0	0	0	Metacarpals	0	0	0	0	0	0	0	0	0	0	0	0	0	0
0	0	0	0	Phalanges digits	0	0	0	0	0	0	0	0	0	0	0	0	0	0
0	0	0	0	Sternum	0	0	0	0	0	0	0	0	0	0	0	0	0	1
0	0	0	0	Osmosternum	0	0	0	0	0	0	0	0	0	0	0	0	0	1
				**Hind Limb**														
1	1	1	1	Femur	1	1	1	1	1	1	1	1	1	1	1	1	1	1
1	1	1	1	Tibia	1	1	1	1	1	1	1	1	1	1	1	1	1	1
1	1	1	1	Fibula	1	1	1	1	1	1	1	1	1	1	1	1	1	1
1	1	1	1	Fibulare	1	1	1	1	1	1	1	1	1	1	1	1	1	1
1	1	1	1	Tibiale	1	1	1	1	1	1	1	1	1	1	1	1	1	1
0	0	0	0	Metatarsalia	0	0	0	0	0	0	0	0	0	0	0	0	0	0
1	1	1	1	Ilium	1	1	1	1	1	1	1	1	1	1	1	1	1	1
0	0	0	0	Ischium	0	0	0	0	0	0	0	0	0	0	0	1	1	1
1	1	1	1	trans processes 1	1	1	1	1	1	1	1	1	1	1	1	1	1	1
1	1	1	1	trans processes 2	1	1	1	1	1	1	1	1	1	1	1	1	1	1
1	1	1	1	trans processes 3	1	1	1	1	1	1	1	1	1	1	1	1	1	1
1	1	1	1	trans processes 4	1	1	1	1	1	1	1	1	1	1	1	1	1	1
1	1	1	1	trans processes 5	1	1	1	1	1	1	1	1	1	1	1	1	1	1
1	1	1	1	trans processes 6	1	1	1	1	1	1	1	1	1	1	1	1	1	1
1	1	1	1	trans processes 7	1	1	1	1	1	1	1	1	1	1	1	1	1	1
1	1	1	1	trans processes 8	1	1	1	1	1	1	1	1	1	1	1	1	1	1
