# Supplementary material for: Comparative Postembryonic Skeletal Ontogeny in Two Sister Lineages of Old World Tree Frogs (Rhacophoridae: Taruga, Polypedates)
Source: PLoS One. 2017 Jan 6;12(1):e0167939. doi: 10.1371/journal.pone.0167939 (PMC5218391; doi:10.1371/journal.pone.0167939)
Supplement: S4 Table — (PDF) [file pone.0167939.s004.pdf]

Skull Bones	25	26	27	28	29	30	31	32	33	34	35	36	37	38	39	40	41	42	43	44	45	46
Exoccipital	0	0	1	1	1	1	1	1	1	1	1	1	1	1	1	1	1	1	1	1	1	1
Parasphenoid	0	0	1	1	1	1	1	1	1	1	1	1	1	1	1	1	1	1	1	1	1	1
Frontoparietal	0	0	0	1	1	1	1	1	1	1	1	1	1	1	1	1	1	1	1	1	1	1
Prootic	0	0	0	0	0	0	0	1	1	1	1	1	1	1	1	1	1	1	1	1	1	1
Septomaxilla	0	0	0	0	0	0	0	0	0	0	0	0	0	0	0	0	0	1	1	1	1	1
Premaxilla	0	0	0	0	0	0	0	0	0	0	0	0	0	0	0	0	1	1	1	1	1	1
Maxilla	0	0	0	0	0	0	0	0	0	0	0	0	0	0	0	0	0	1	1	1	1	1
Nasal	0	0	0	0	0	0	0	0	0	0	0	0	0	0	0	0	0	1	1	1	1	1
Squamosal	0	0	0	0	0	0	0	0	0	0	0	0	0	0	0	0	0	0	1	1	1	1
Angulosplenia	0	0	0	0	0	0	0	0	0	0	0	0	0	0	0	0	0	1	1	1	1	1
Vomer	0	0	0	0	0	0	0	0	0	0	0	0	0	0	0	0	0	0	0	0	1	1
Dentary	0	0	0	0	0	0	0	0	0	0	0	0	0	0	0	0	0	0	1	1	1	1
Pterygoid	0	0	0	0	0	0	0	0	0	0	0	0	0	0	0	0	0	0	1	1	1	1
Quadratojugal	0	0	0	0	0	0	0	0	0	0	0	0	0	0	0	0	0	0	0	1	1	1
Columella	0	0	0	0	0	0	0	0	0	0	0	0	0	0	0	0	0	0	0	0	1	1
Mentomeckelian	0	0	0	0	0	0	0	0	0	0	0	0	0	0	0	0	0	0	0	0	0	1
Palatine	0	0	0	0	0	0	0	0	0	0	0	0	0	0	0	0	0	0	0	1	1	1
Teeth	0	0	0	0	0	0	0	0	0	0	0	0	0	0	0	0	0	0	0	0	1	1
Sphenethmiod	0	0	0	0	0	0	0	0	0	0	0	0	0	0	0	0	0	0	0	0	0	0
**Hyobranchial**																						
Hyoid	0	0	0	0	0	0	0	0	0	0	0	0	0	0	0	0	0	0	0	0	0	0
**Axial**																						
Neural arch 1	0	0	0	0	1	1	1	1	1	1	1	1	1	1	1	1	1	1	1	1	1	1
Neural arch 2	0	0	0	0	1	1	1	1	1	1	1	1	1	1	1	1	1	1	1	1	1	1
Neural arch 3	0	0	0	0	1	1	1	1	1	1	1	1	1	1	1	1	1	1	1	1	1	1
Neural arch 4	0	0	0	0	0	1	1	1	1	1	1	1	1	1	1	1	1	1	1	1	1	1
Neural arch 5	0	0	0	0	0	0	1	1	1	1	1	1	1	1	1	1	1	1	1	1	1	1
Neural arch 6	0	0	0	0	0	0	0	0	0	1	1	1	1	1	1	1	1	1	1	1	1	1
Neural arch 7	0	0	0	0	0	0	0	0	0	0	1	1	1	1	1	1	1	1	1	1	1	1
Neural arch 8	0	0	0	0	0	0	0	0	0	0	1	1	1	1	1	1	1	1	1	1	1	1
sacral	0	0	0	0	0	0	0	0	0	0	0	1	1	1	1	1	1	1	1	1	1	1
Centra1	0	0	0	0	0	0	0	0	0	1	1	1	1	1	1	1	1	1	1	1	1	1
Centra2	0	0	0	0	0	0	0	0	0	1	1	1	1	1	1	1	1	1	1	1	1	1
Centra3	0	0	0	0	0	0	0	0	0	1	1	1	1	1	1	1	1	1	1	1	1	1
Centra4	0	0	0	0	0	0	0	0	0	0	1	1	1	1	1	1	1	1	1	1	1	1
Centra5	0	0	0	0	0	0	0	0	0	0	1	1	1	1	1	1	1	1	1	1	1	1
Centra6	0	0	0	0	0	0	0	0	0	0	0	1	1	1	1	1	1	1	1	1	1	1
Centra7	0	0	0	0	0	0	0	0	0	0	0	1	1	1	1	1	1	1	1	1	1	1
Centra8	0	0	0	0	0	0	0	0	0	0	0	0	1	1	1	1	1	1	1	1	1	1
sacral diapophysis	0	0	0	0	0	0	0	0	0	0	0	0	0	0	0	1	1	1	1	1	1	1
Hypochord	0	0	0	0	0	0	0	0	0	0	0	0	0	0	0	1	1	1	1	1	1	1
Coccyx	0	0	0	0	0	0	0	0	0	0	0	0	0	0	0	0	0	0	0	0	0	0
UROSTYLE	0	0	0	0	0	0	0	0	0	0	0	0	0	1	1	1	1	1	1	1	1	1
**Fore limbs**																						
Scapula	0	0	0	0	0	0	0	0	0	0	1	1	1	1	1	1	1	1	1	1	1	1
Humerus	0	0	0	0	0	0	0	0	0	0	1	1	1	1	1	1	1	1	1	1	1	1
Ulna	0	0	0	0	0	0	0	0	0	0	1	1	1	1	1	1	1	1	1	1	1	1

	25	26	27	28	29	30	31	32	33	34	35	36	37	38	39	40	41	42	43	44	45	46
Radius	0	0	0	0	0	0	0	0	0	0	1	1	1	1	1	1	1	1	1	1	1	1
Cleithrum	0	0	0	0	0	0	0	0	0	0	1	1	1	1	1	1	1	1	1	1	1	1
Clavicle	0	0	0	0	0	0	0	0	0	0	0	1	1	1	1	1	1	1	1	1	1	1
Coracoid	0	0	0	0	0	0	0	0	0	0	0	1	1	1	1	1	1	1	1	1	1	1
Metacarpals	0	0	0	0	0	0	0	0	0	0	0	1	1	1	1	1	1	1	1	1	1	1
Phalanges digits	0	0	0	0	0	0	0	0	0	0	0	0	0	1	1	1	1	1	1	1	1	1
Sternum	0	0	0	0	0	0	0	0	0	0	0	0	0	0	0		0	0	0	0	0	1
Osmosternum	0	0	0	0	0	0	0	0	0	0	0	0	0	0	0		0	0	0	0	0	1
**Hind Limb**																						
Femur	0	0	0	0	0	0	0	0	0	0	0	0	1	1	1	1	1	1	1	1	1	1
Tibia	0	0	0	0	0	0	0	0	0	0	0	0	1	1	1	1	1	1	1	1	1	1
Fibula	0	0	0	0	0	0	0	0	0	0	0	0	1	1	1	1	1	1	1	1	1	1
Fibulare	0	0	0	0	0	0	0	0	0	0	0	0	1	1	1	1	1	1	1	1	1	1
Tibiale	0	0	0	0	0	0	0	0	0	0	0	0	1	1	1	1	1	1	1	1	1	1
Metatarsalia	0	0	0	0	0	0	0	0	0	0	0	0	1	1	1	1	1	1	1	1	1	1
Ilium	0	0	0	0	0	0	0	0	0	0	0	0	1	1	1	1	1	1	1	1	1	1
Ischium	0	0	0	0	0	0	0	0	0	0	0	0	0	1	1	1	1	1	1	1	1	1
trans processes 1					1	1	1	1	1	1	1	1	1	1	1	1	1	1	1	1	1	1
trans processes 2							1	1	1	1	1	1	1	1	1	1	1	1	1	1	1	1
trans processes 3							1	1	1	1	1	1	1	1	1	1	1	1	1	1	1	1
trans processes 4							0	1	1	1	1	1	1	1	1	1	1	1	1	1	1	1
trans processes 5							0	0	0	0	0	0	0	1	1	1	1	1	1	1	1	1
trans processes 6							0	0	0	0	0	0	0	0	0	0	1	1	1	1	1	1
trans processes 7							0	0	0	0	0	0	0	0	0	0	1	1	1	1	1	1
trans processes 8							0	0	0	0	0	0	0	0	0	0	1	1	1	1	1	1
