# Supplementary material for: Comparative Postembryonic Skeletal Ontogeny in Two Sister Lineages of Old World Tree Frogs (Rhacophoridae: Taruga, Polypedates)
Source: PLoS One. 2017 Jan 6;12(1):e0167939. doi: 10.1371/journal.pone.0167939 (PMC5218391; doi:10.1371/journal.pone.0167939)
Supplement: S5 Table — (PDF) [file pone.0167939.s005.pdf]

Table S5.

Genus	species	Accession numbers	Location	Accession no.
Taruga	*eques*	WHT2714	Sri Lanka	AY141846
Taruga	*eques*	WHT2741	Sri Lanka	AY141847
Taruga	*eques* (TP)	DZ 1613	Sri Lanka	to be submitted
Taruga	*fastigo*	WHT2783	Sri Lanka	AY141848
Taruga	*longinasus*	WHTKAN1	Sri Lanka	GQ204691
Taruga	*longinasus* (TP)	DZ 1614	Sri Lanka	to be submitted
Polypedates	*cruciger*	WHTKuru3	Sri Lanka	GQ204692
Polypedates	*cruciger*	WHT2640	Sri Lanka	GQ204687
Polypedates	*cruciger* (TP)	DZ 1611	Sri Lanka	to be submitted
Polypedates	*maculatus* (TP)	DZ 1612	Sri Lanka	to be submitted
Polypedates	*maculatus*	WHTKANT	Sri Lanka	GQ204694
Polypedates	*leucomystax varB*	GenBank	Java	GQ204698
Polypedates	*colletti*	FMNH242765	Malaysia	GQ204697
Polypedates	*leucomystax*	FMNH255296	Vietnam	GQ204700
Polypedates	*leucomystax*	FMNH256451	Laos	GQ204701
Polypedates	*leucomystax*	ZRC1.1.5269	Laos	GQ204693
Polypedates	*leucomystax var A*	FMNH253086	Vietnam	GQ204699
Polypedates	*macrotis*	FMNH239119	Malaysia	GQ204695
Polypedates	*otilophus*	FMNH239147	Malaysia	GQ204696
Rhacophorus	*anamensis*	FMNH253934	Vietnam	GQ204717
Rhacophorus	*bipunctatus*	FMNH253114	Vietnam	GQ204716
Rhacophorus	*calcaneus*	FMNH256465	Laos	GQ204719
Rhacophorus	*chenfui*	FMNH232964	China	GQ204712
Rhacophorus	*dulitensis*	FMNH235741	Malaysia	GQ204715
Rhacophorus	*gauni*	FMNH235047	Malaysia	GQ204714
Rhacophorus	*malabaricus*	GenBank	India	AF249050
Rhacophorus	*nigropalmatus*	FMNH230902	Malaysia	GQ204710
Rhacophorus	*pardalis*	FMNH231366	Malaysia	GQ204711
Rhacophorus	*reinwardtii*	ZRC1.1.5273	Java	GQ204720
Rhacophorus	*reinwardtii*	FMNH235034	Malaysia	GQ204713
Rhacophorus	*sp.*	FMNH255280	Laos	GQ204718
Ghatixalus	*variabilis*	SDB2010.275	India	KR259639
Ghatixalus	*variabilis*	SDB4990	India	KR259640
Mantella	*aurantiaca*	GenBank	Madagascar	DQ283035
Mantella	*madagascariensis*	GenBank	Madagascar	AF249049
Mantella	*sp.*	GenBank	Madagascar	AF026372
Mantidactylus	*grandidieri*	GenBank	Madagascar	AY341712
